# Characterization of Blood Immune Cells in Patients With Decompensated Cirrhosis Including ACLF

**DOI:** 10.3389/fimmu.2020.619039

**Published:** 2021-02-05

**Authors:** Emmanuel Weiss, Pierre de la Grange, Mylène Defaye, Juan José Lozano, Ferrán Aguilar, Pushpa Hegde, Ariane Jolly, Lucile Moga, Sukriti Sukriti, Banwari Agarwal, Haqeeqat Gurm, Marion Tanguy, Johanne Poisson, Joan Clària, Paer-Selim Abback, Axel Périanin, Gautam Mehta, Rajiv Jalan, Claire Francoz, Pierre-Emmanuel Rautou, Sophie Lotersztajn, Vicente Arroyo, François Durand, Richard Moreau

**Affiliations:** ^1^Assistance Publique—Hôpitaux de Paris (AP-HP), Department of Anesthesiology and Critical Care, Beaujon Hospital, DMU Parabol, AP-HP Nord, Paris, France; ^2^Université de Paris, Institut National de la Santé et de la Recherche Médicale (INSERM), Centre de Recherche sur l’Inflammation (CRI), Paris, France; ^3^European Foundation for the study of Chronic Liver Failure (EF-Clif), European Association for the Study of Chronic Liver Failure (EASL-CLIF) Consortium and Grifols Chair, Barcelona, Spain; ^4^GenoSplice, Paris, France; ^5^CIBERehd, Barcelona, Spain; ^6^Assistance Publique—Hôpitaux de Paris (APHP), Service d’Hépatologie & Réanimation Hépato Digestive, Hôpital Beaujon, Clichy, France; ^7^Department of Research, Institute of Liver and Biliary Sciences, New Delhi, India; ^8^Liver Failure Group, Institute for Liver and Digestive Health, University College London, London, United Kingdom; ^9^Hospital Clínic-August Pi i Sunyer Biomedical Research Institute (IDIBAPS), Barcelona, Spain; ^10^Universitat de Barcelona, Barcelona, Spain; ^11^Institute of Hepatology, Foundation for Liver Research, London, United Kingdom; ^12^Faculty of Life Sciences & Medicine, King’s College London, London, United Kingdom

**Keywords:** myeloid cells, innate lymphoid cells, adaptive immune cells, sepsis, organ failure, immunotherapies

## Abstract

**Background and Aims:**

Patients with cirrhosis and acute-on-chronic liver failure (ACLF) have immunosuppression, indicated by an increase in circulating immune-deficient monocytes. The aim of this study was to investigate simultaneously the major blood-immune cell subsets in these patients.

**Material and Methods:**

Blood taken from 67 patients with decompensated cirrhosis (including 35 critically ill with ACLF in the intensive care unit), and 12 healthy subjects, was assigned to either measurements of clinical blood counts and microarray (genomewide) analysis of RNA expression in whole-blood; microarray (genomewide) analysis of RNA expression in blood neutrophils; or assessment of neutrophil antimicrobial functions.

**Results:**

Several features were found in patients with ACLF and not in those without ACLF. Indeed, clinical blood count measurements showed that patients with ACLF were characterized by leukocytosis, neutrophilia, and lymphopenia. Using the CIBERSORT method to deconvolute the whole-blood RNA-expression data, revealed that the hallmark of ACLF was the association of neutrophilia with increased proportions of macrophages M0-like monocytes and decreased proportions of memory lymphocytes (of B-cell, CD4 T-cell lineages), CD8 T cells and natural killer cells. Microarray analysis of neutrophil RNA expression revealed that neutrophils from patients with ACLF had a unique phenotype including induction of glycolysis and granule genes, and downregulation of cell-migration and cell-cycle genes. Moreover, neutrophils from these patients had defective production of the antimicrobial superoxide anion.

**Conclusions:**

Genomic analysis revealed that, among patients with decompensated cirrhosis, those with ACLF were characterized by dysregulation of blood immune cells, including increases in neutrophils (that had a unique phenotype) and macrophages M0-like monocytes, and depletion of several lymphocyte subsets (including memory lymphocytes). All these lymphocyte alterations, along with defective neutrophil superoxide anion production, may contribute to immunosuppression in ACLF, suggesting targets for future therapies.

## Introduction

Acute-on-chronic liver failure (ACLF), which develops in patients with acutely decompensated cirrhosis, is characterized by the existence of organ failure(s) and high in-hospital mortality (1, 2). ACLF is associated with systemic inflammation as indicated by blood leukocytosis (1, 2), and high plasma levels of C-reactive protein (1, 2) and cytokines and chemokines (3, 4). White blood-cell count is a component of the chronic liver failure consortium ACLF scoring system, which accurately predicts early mortality in patients with ACLF (5). It has been suggested that, in ACLF, peripheral leukocytosis is enriched in effector immune cells that have a high potential to cause tissue damage (2). ACLF is also characterised by systemic inflammation which is known to be energetically expensive and may thus skew nutrients otherwise required for other metabolic processes towards immune cells. This would therefore deny peripheral organs the required nutrients which may result in systemic organ failure (6).

There are studies concentrating on peripheral-blood myeloid mononuclear cells involved in innate immunity (4, 7–11), showing, for example, that patients with ACLF, whether critically ill or not, have increased frequency of CD14^+^ monocytes expressing the receptor tyrosine kinase MerTK (hereafter called MerTK) (4, 10) and CD14^+^CD15^-^HLA-DR^-^ myeloid-derived suppressor cells (9). Both subsets of myeloid mononuclear cells have suppressed innate responses to bacterial pathogen-associated molecular patterns (PAMPs) (4, 9, 10). These alterations may, therefore, favor the development of bacterial infections that are frequent complications of ACLF (12, 13). Another study found decreased frequencies of other myeloid mononuclear cells (conventional dendritic cells [DCs] and plasmacytoid DCs) in patients with severe alcoholic hepatitis, including patients who had ACLF (11). Several studies have showed alterations in neutrophils (14–16) and lymphocytes (7, 17–19) in patients with decompensated cirrhosis. However, until now there has been no comprehensive description of the landscape of blood immune-cell subsets in patients with ACLF, in particular critically ill patients admitted to the intensive care units (ICUs) and who have an especially poor prognosis. A better knowledge of circulating immune-cell landscape in patients with ACLF could provide new insights into the pathophysiology of systemic inflammation and organ failures and identify targets for therapies, which are unmet medical needs (2). We therefore aimed to perform a pilot study with the objective of characterising white blood cell subsets beyond just monocytes and dendritic cells, in critically ill patients with ACLF, patients with decompensated cirrhosis but without ACLF and in healthy subjects (HS).

To address this question, we performed microarray (genomewide) analysis of RNA expression in whole-blood from study individuals. Indeed, a large number of information about a variety of circulating immune-cell subsets have been identified from genomewide analysis of RNA expression in a single blood sample obtained in each study individual (20–23).

## Material and Methods

### Patients

The core study was a pilot aiming to investigate clinical complete blood counts and whole-blood transcriptome in patients with biopsy-proven cirrhosis admitted to the Hepatology and Liver Intensive Care Unit at Beaujon Hospital, Clichy, France (**Table S1**). The study protocol was approved by the local human research ethics committee (Supporting Methods). Written informed consent before enrollment or delayed consent was obtained from each patient, or legal surrogate. Every patient with ACLF was admitted to the ICU for organ support; blood used for whole-blood transcriptomics was taken within 24 h after admission to the ICU. We used criteria developed by the European Foundation for the Study of Chronic Liver Failure for the diagnosis of acutely decompensated cirrhosis, organ failures, and ACLF grades (**Table S2**) (1, 5, 24).

Of the 31 patients who were enrolled in the core pilot study, 24 were nonelectively admitted to the hospital [including 17 critically ill patients with ACLF (94% of them having circulatory failure), and seven who had acutely decompensated cirrhosis without ACLF (a group hereafter called AD)], and seven were admitted for therapeutic paracentesis of refractory ascites [a group hereafter called advanced cirrhosis (AC)] (**Table 1**). Of note, patients with AC were stable; indeed, none of the patients with AC had ongoing or recently treated (less than 1 week) bacterial infection or gastrointestinal hemorrhage. During the 90 days following enrollment, the number of patients who received a liver transplant was 4, 3, and 3, in the ACLF, AD, and AC groups, respectively. The 90-day transplant-free mortality rate was 71%, 14%, and 0%, in the ACLF, AD, and AC groups, respectively.

**Table 1:** Characteristics of the French patients with cirrhosis enrolled for the whole-blood transcriptome analysis.

Characteristic		Advanced cirrhosis (AC, n=7)		Acute decompensation of cirrhosis (AD*^a^*, n=7)	ACLF (n=17)			P value for the difference between all groups		P value for the difference between AC and AD		P value for the difference between ACLF and AD	
Median age (IQR) − yr		59 (53–64)		57 (52–59)	58 (53–60.5)			0.81		−		−	
Male sex – n (%)		7 (100)		3 (42.9)	13 (76.5)			0.06		−		−	
Etiology or cirrhosis – n (%)								0.45		–		–	
Alcoholic liver disease		6 (86)		3 (43)	12 (70)								
Nonalcoholic steatohepatitis		1 (14)		2 (28)	3 (18)								
Chronic hepatitis C		0 (0)		1 14)	2 (12)								
Chronic hepatitis B		0 (0)		1 (14)	0 (0)								
Acute precipitants – n (%)								−		−		0.66	
Sepsis		0 (0)		2 (28.6)	8 (47.1)								
Excessive alcohol consumption		0 (0)		2 (28.6)	1 (5.9)								
Hemorrhage		0 (0)		1 (14.3)	4 (23.5)								
Other		0 (0)		1 (14.3)	2 (11.8)								
None		7 (100)		1 (14.3)	2 (11.8)								
Severity scores													
Median CLIF-C AD score (IQR)		–		55 (52–59)	–			–		–		–	
Median MELD score (IQR)		10 (8–18)		21 (15–25)	35 (26–39)			<0.001		0.05		0 .003	
Median CLIF-C Organ Failure score (IQR)		–		8 (7–9)	14 (12–15)			–		–		<0.001	
Median CLIF-C ACLF score (IQR)		–		–	67 (59–69.5)			–		–		–	
Individual organ system failures*^a^* – n (%)												
Kidney		0 (0)		0 (0)		11 (64.7)	<0.001			1		0.005	
Lungs		0 (0)		0 (0)		10 (59.0)	0.003			1		0.02	
Liver		0 (0)		1 (14.3)		4 (23.5)	0.37			−		−	
Coagulation		0 (0)		1 (14.3)		11 (64.7)	0.005			0.71		0.05	
Brain		0 (0)		0 (0)		3 (17.6)	0.26			−		−	
Circulation		0 (0)		0 (0)		16 (94.0)	<0.001			1		<0.001	
ACLF Grade*^a^* – n (%)							–			–		–	
Grade 1		0 (0)		0 (0)	1 (5.9)								
Grade 2		0 (0)		0 (0)	2 (11.8)								
Grade 3		0 (0)		0 (0)	14 (82.4)								
Median white-cell count*^b^* (IQR) − per mm^3^		6,000 (4,400–8,300)		5,600 (4,500–8,700)	16,100 (10,200–22,200)			0.01		0.8		0.02	
Median absolute count (IQR) − per mm^3^													
Neutrophils		4,250 (2595–4940)		3,680 (2,900–4,795)	12,800 (7,700–18,400)			0.022		0.9		0.013	
Monocytes		700 (580–1,090)		1,000 (690–1,100)	1,100 (600–1,490)			0.28		–		–	
Lymphocytes		1,300 (1,045–1,785)		1,000 (925–1,535)	1,080 (700–1,600)			0.95		–		–	
Median differential count*^c^* (IQR) − %													
Neutrophils		66 (46–72)		60 (56–66)	81 (70–90)			<0.001		0.53		<0.001	
Lymphocytes		18 (15–3)		18 (13–27)	11 (4–16)			0.008		0.9		0.016	
Monocytes		12 (8–13)		13 (7–22)	9 (4–13)			.14		−		−	
Median hemoglobin (IQR) − g/dl		11.6 (10.1–14.1)		7.8 (7.1–10.1)	8.8 (8.1–11.3)			0.02		0.02		0.166	
Median platelet count (IQR) − per mm^3^		71,000 (65,000–265,000)		80,000 (73,000–157,000)	116,000 (84,500–164,000)			0.79		−		−	
Median International Normalized Ratio (IQR)		1.42 (1.20–1.86)		1.64 (1.54–2.23)	2.80 (2.10–3.10)			<0.001		0.13		0.013	
Median serum sodium (IQR) − mmol/L		135 (133–138)		133 (131–135)	133 (125–137)			0.45		−		−	
Median serum creatinine (IQR) − mg/dl		0.74 (0.68–0.85)		0.73 (0.56–1.51)	2.40 (1.90–3.10)			0.001		0.46		<0.001	
Median C-reactive protein (IQR) − mmol/L		7 (7–10)		11 (7–24)	44 (21–94)			0.36		−		−	
Median aspartate aminotransferase (IQR) − U/L		58 (33–72)		50 (46–90)	104 (61–234)			0.05		−		−	
Median alanine aminotransferase (IQR) − U/L		26 (12–33)		28 (14–37)	40 (20–83)			0.12		−		−	
Median total bilirubin (IQR) − mg/dl		2.47 (0.80–4.71)		5.82 (1.81–6.23)	5.10 (3.20–11.70)			0.10		−		−	
Outcome													
Death without transplantation by 90 days – n (%)		0 (0)		0 (0)	12 (71)			–		–		–	
Liver transplantation – n (%)		3 (43)		3 (43)	4 (23.5)			–		–		–	

P values were obtained using Kruskal–Wallis test and Mann–Whitney test and the chi-square test or Fisher’s exact test, as appropriate. ACLF denotes acute-on-chronic liver failure; IQR, interquartile range; CLIF-C, Chronic Liver Failure-Consortium; and MELD, Model for End Stage Liver Disease.

a Defined in **Table S2**.

b Normal range for blood white-cell count is 4,500–11,000 per mm^3^.

c Normal ranges for differential counts are 40%–70% for neutrophils, 4%–11% for monocytes, and 22%–44% for lymphocytes.

### Methods

Methods which are described in detail in the Supporting Information, are summarized as follows.

#### Microarray (Genomewide) Analysis of Whole-Blood RNA Expression

Blood was collected in Tempus tubes (Thermo Fisher Scientific, Waltham, MA). RNA was extracted using the 5 PRIME PerfectPure RNA blood kit (Thermo Fisher Scientific) according to manufacturer’s instructions. One hundred nanograms of RNA per sample were then hybridized on Human Transcriptome Array 2.0 (HTA2.0, Affymetrix, Santa Clara, CA) at the genomic platform of the Curie Institute (Paris, France). This array was designed to measure the RNA expression of 67,528 genes. Analysis and visualization of the Human Transcriptome Array 2.0 dataset were made using EASANA^®^ (GenoSplice technology), which was based on the GenoSplice’s FAST DB^®^ annotations (18). Expression data were log_2_-transformed and used for two purposes. First, Student’s *t-*test were used for identification differentially expressed genes (DEGs). Genes with a fold change >1.5-fold and P <0.05 were considered as differential expressed. Then, we performed enrichment analysis using as gene sets, the 346 blood transcription modules (hereafter referred to as BTMs) that have been developed by Li, Rouphael, Duraisingham, et al (21). and were publicly available (**Table S3**). These BTMs have been computed using transcriptomic data obtained in peripheral-blood mononuclear cells from individuals under various immunological stimuli (21). Each BTM contained a variable number of co-expressed genes and has received an identification number. A large number of BTMs were related to specific immune-cell subsets; for example, the BTMs “M.37.1: enriched in neutrophils (I)”; “M11.0: enriched in monocytes (II); “M7.0: Enriched in T cells (I)”; “M7.2: enriched in NK cells (I)”; “M47.0: enriched in B cells (I)”. Other modules were related to immune signaling; for example, “M16: TLR and inflammatory signaling”; M37.0: immune activation - generic cluster” (**Table S3**). Enrichment analysis also used other publicly available data bases such as Reactome pathways (https://reactome.org) as gene sets.

Second, the CIBERSORT software package was used for deconvoluting the blood RNA microarray data (22). R values were calculated to assess correlations of complete blood counts inferred from CIBERSORT and counts measured by clinical laboratories. Inferred CIBERSORT lymphocyte counts were calculated as the sum of the counts of naive B cells, memory B cells, CD8 T cells, naive CD4 T cells, resting memory CD4 T cells, and activated memory CD4 T cells (23). The counts of monocytes, macrophage M0- and macrophage M2-like monocytes were summed to infer CIBERSORT monocyte counts (23).

#### Microarray (Genomewide) Analysis of RNA Expression in Blood Neutrophils

RNA was extracted from freshly isolated neutrophils. One hundred nanograms of RNA per sample were then hybridized on Clariom S Array, Human (Affymetrix) at the genomic platform of the Curie Institute (Paris, France). This array was designed to measure the RNA expression of ~20,800 genes.

## Results

### ACLF Associates Blood Leukocytosis, Neutrophilia, and Lymphopenia

**Figure 1:**
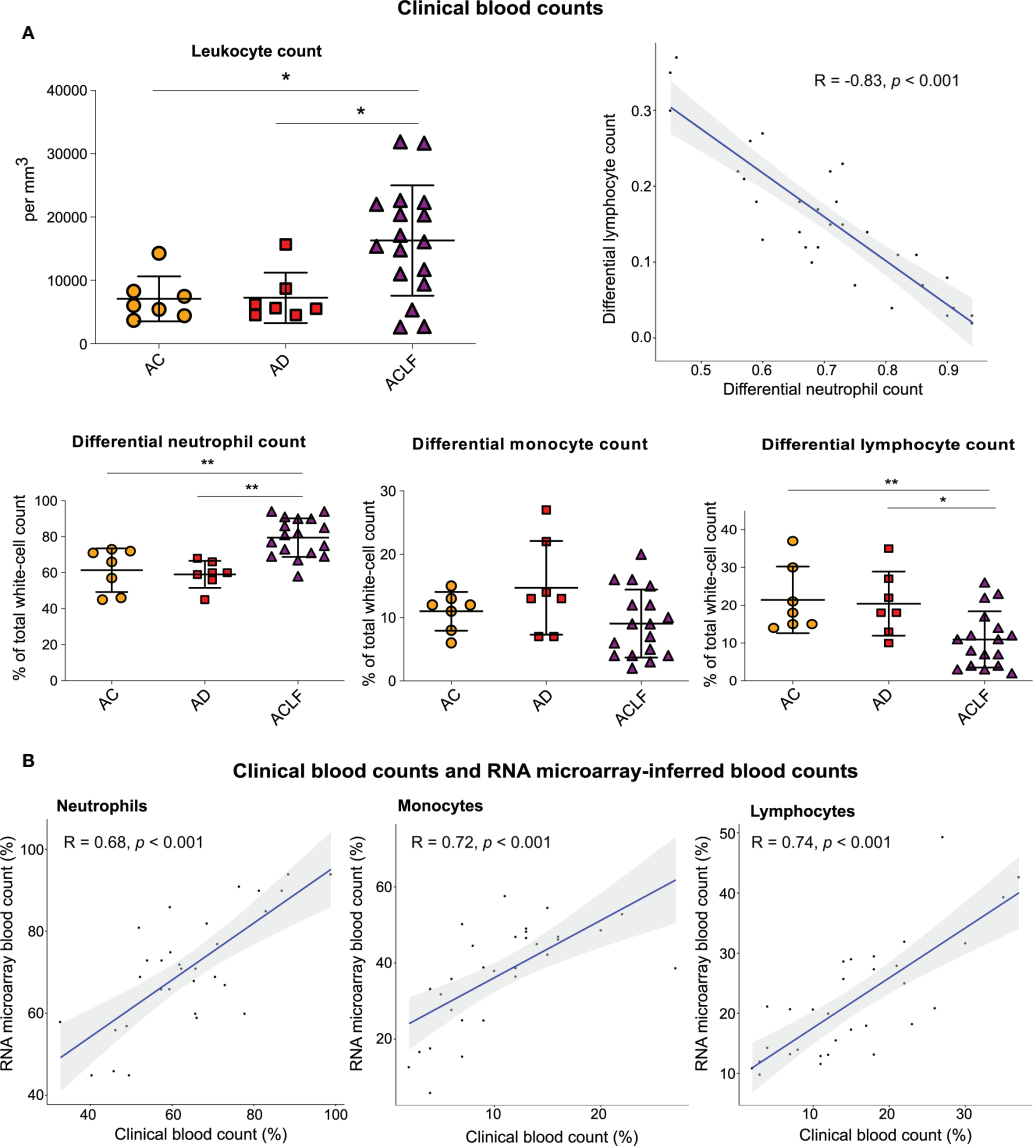
Results of clinical blood counts and RNA microarray-inferred blood counts. **(A)** Clinical complete blood counts in three groups of patients with cirrhosis: AC (orange circles, n=7), AD (red square, n=7) and ACLF (purple triangle, n=17). P values were obtained using *Kruskal–Wallis test* and Mann–Whitney test. **P <0.01; *P <0.05. **(B)** Neutrophil, lymphocyte, and monocyte counts in paired clinical complete blood counts as compared with the CIBERSORT-inferred blood counts from RNA microarray data obtained with the use of peripheral blood (31 paired specimens). The shaded areas represent the 95% confidence intervals. AC denotes advanced cirrhosis, AD acute decompensation, and ACLF acute on chronic liver failure.

Clinical complete blood counts obtained on admission in the 31 French patients with cirrhosis showed that ACLF was characterized by significant increases in white-cell count (i.e., leukocytosis), differential and absolute neutrophil counts (i.e., neutrophilia), and significant decreases in differential lymphocyte count (i.e., lymphopenia), as compared with the two other groups (AC and AD) (**Figure 1A**, **Figure S1**). Absolute lymphocyte count, and monocyte counts (differential and absolute) did not significantly differ between ACLF and the two other groups (**Figure 1A**, **Figure S1**). No significant differences in blood counts were seen between AC and AD.

Similar findings (**Figures S2A, B**) were obtained in an independent English cohort including 91 patients with AD and 203 critically ill patients with ACLF (Supplementary Methods; **Table S4**). In both, the French cohort (**Figure 1A**) and the English cohort (**Figure S2C**), there was a significant negative correlation between differential neutrophil count and differential lymphocyte count, indicating a dichotomic regulation of circulating immune cells, opposing neutrophils to lymphocytes, and culminating in ACLF.

### Validation of the CIBERSORT-Inferred Blood Counts

We found that the RNA microarray–inferred white-cell counts (calculated based on the results of the CIBERSORT method; **Table S5**) **(**23) were positively correlated with clinical laboratory measurements of complete blood counts (**Figure 1B**), which suggested that microarray analysis of whole-blood RNA was of sufficient quality to provide information that correlated with criterion-standard clinical measurements of blood counts.

### RNA Identification of Dysregulated Blood Immune-Cell Subsets in ACLF

Genomewide analysis of whole-blood RNA expression (microarrays) was performed in the 31 patients from the French cohort and 7 HS (healthy subjects) (**Table S1**). Unsupervised hierarchical clustering analysis of the results showed that the profiles of blood RNA expression were markedly different between patients with ACLF and all the other study persons (**Figure S3**). Microarray results were validated by using RT-qPCR in a random set of genes (**Figure S4**).

**Figure 2:**
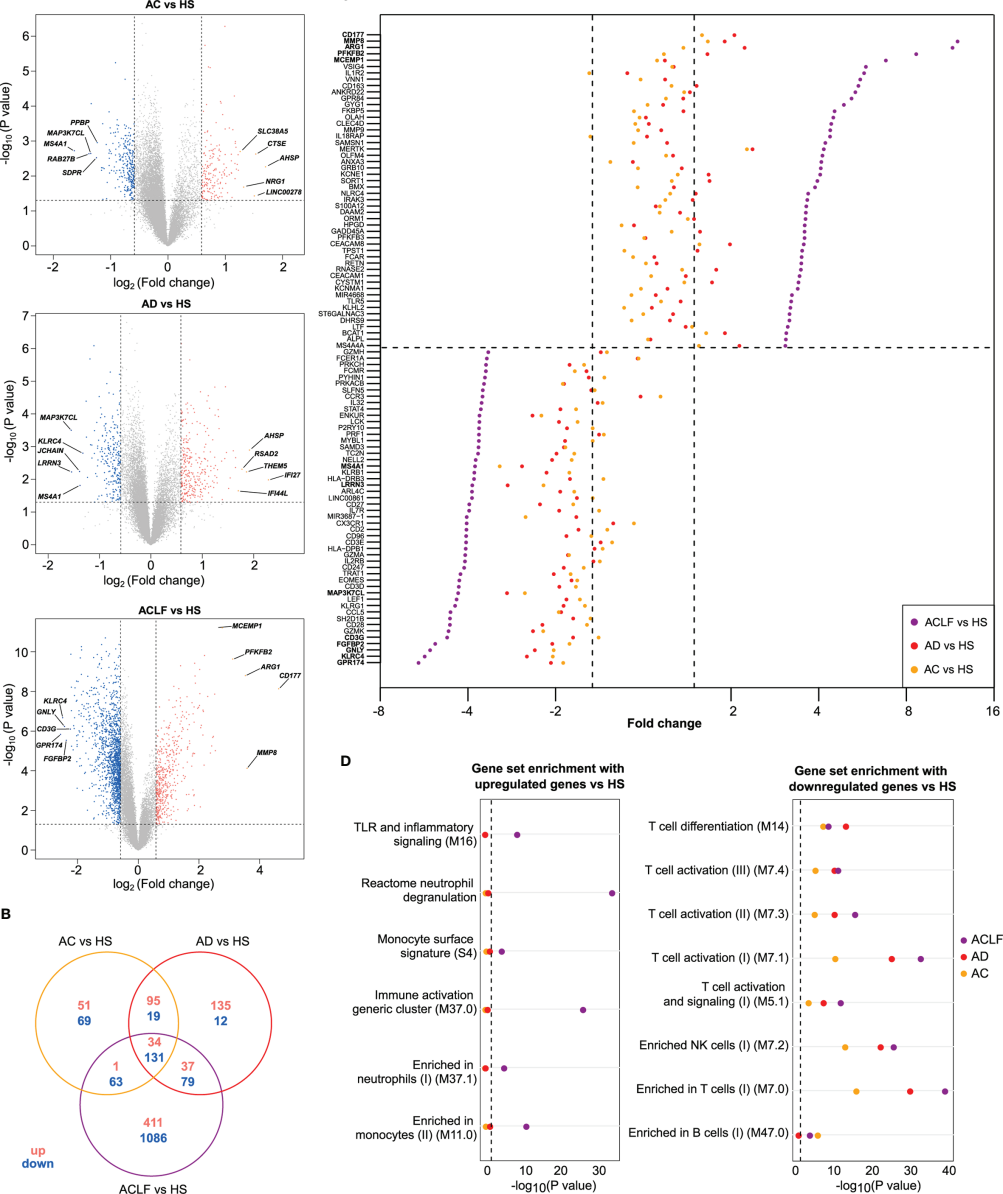
Distinctive transcriptional characteristics of ACLF. **(A)** Volcano plots of differential gene expression between each patients’ group [AC (n=7); AD (n=7); ACLF (n=17)] relative to healthy subjects [HS (n=7)], with significance (−log_10_ P value) plotted against the log_2_ fold-change (patients:HS ratio). Genes were considered as differentially expressed when P was <0.05 (or −log_10_ P >1.3; dashed horizontal line) and fold-change >1.5 [or log_2_ fold-change >0.58 for upregulation or log_2_ fold-change <-0.58 for downregulation (right and left dashed vertical lines, respectively)]. Gray points indicate genes with no significant difference in expression, salmon indicates genes with significantly increased expression in patients and blue indicates genes with significantly decreased expression in patients. Representative differentially expressed genes are shown. CD177 was the most upregulated gene in ACLF versus HS. Between-group comparisons were performed using Student’s *t*-test. **(B)** Venn diagram showing the number of up- and downregulated genes that were unique or not to each of the three pairwise comparisons. **(C)** Cleveland plot showing the fold-changes for the 50 most upregulated genes in ACLF versus HS (top) and in the 50 most downregulated in this comparison (bottom). The dashed horizontal line separates upregulated genes from down regulated genes. For each gene, the fold-change of expression is also shown for two other pairwise comparisons: AD versus HS and AC versus HS. A dashed vertical line (right) indicates the threshold of 1.5 fold-change versus HS and the other dashed vertical line (left) indicates the threshold of -1.5 fold-change versus HS. **(D)** Results of enrichment analysis of predefined data sets (which, unless specified, were blood transcription modules (21)) with upregulated genes (left) and downregulated genes (right), both in ACLF versus HS. The enrichment of these gene sets with genes that were upregulated (left) or downregulated (right), in AC versus HS and AD versus HS genes, are also shown.

Then, the results of microarray analysis were used to establish the lists of DEGs, corresponding to 3 pairwise comparisons: AC versus HS, AD versus HS, and ACLF versus HS (hereafter referred to as AC/HS, AD/HS, and ACLF/HS, respectively; **Table S6**), that were visualized using volcano plots (**Figure 2A**). In AC/HS (**Figure 2A**, left), the 5 top upregulated genes included *SCLC38A5, CTSE, AHSP, NRG1, LINC00278* while the 5 top downregulated genes included *PPBP*, *MAP3K7CL, MS4A1, RAB27B, SDPR*. In AD/HS (**Figure 2A**, middle), the top upregulated genes included *AHSP, RSAD2, IFI27, IFI44L, THEM5* while the top downregulated genes included *MAP3K7CL, KLRC4, JCHAIN, MS4A1, LRNN3*. In ACLF/HS (**Figure 2A**, right), the top upregulated genes included *CD177, MCEMP1, ARG1, MMP9, PFKFB2* while the top downregulated genes included *CD3G, KLRC4, GNLY, FGFBP2, GPR174*. Of note, in ACLF/AC the 5 top upregulated genes (**Figure S5**; **Table S6**) were similar to the corresponding top upregulated genes in ACLF/HS (**Figure 2A**, right). In ACLF/AC, the only top downregulated gene shared with ACLF/HS was *CD3G*. In ACLF/AC, the other top downregulated genes were *FCER1A, CCR3, CLC, CX3CR1* (**Figure S5**; **Table S6**).

In addition, we used Venn diagrams to visualize the number of DEGs unique to each comparison. There were few DEGs unique to AC/HS or AD/HS (120 and 147, respectively) while there were 1497 DEGs unique to ACLF/HS, indicating changes in blood transcriptome that were specific for ACLF (**Figure 2B**).

#### ACLF Associates With Increased Innate Inflammatory Signatures in Blood

To gain insights into the dynamics of transcription at the single-gene level across the study groups, we first used the comparison of ACLF versus HS to rank fold-changes in gene expression, from the highest to the lowest fold-change value, and then aligned, for each gene, its fold-change values in the two other pairwise comparisons (AC/HS, AD/HS; **Table S6**). Using the fold-changes values for the 50 most upregulated genes in ACLF/HS, and the corresponding values in the two other comparisons (**Table S6**), we drew a Cleveland plot showing fold-changes for each gene across the three pairwise comparisons (**Figure 2C**, top). For each gene, the fold-change was higher in ACLF/HS than the fold-change in the two other comparisons, and, in both AC/HS and AD/HS, very few genes passed the filter defining significant upregulation (**Figure 2C**, top; **Table S6**). We also noted that 22 of the 50 most upregulated genes in ACLF/HS were related to innate immunity; for example *CD177* [that had the highest expression (**Figures 2A, C****)**], *MMP8*, *MMP9, ARG1, S100A12*, were neutrophil genes. Together, these findings suggested that upregulated genes in ACLF/HS composed a distinctive gene signature of ACLF. Then, the BTM gene modules (**Table S3**) **(**21) and canonical pathways (in particular, Reactome pathways) were used as gene sets to perform enrichment analysis of the three complete lists of upregulated genes (ACLF/HS, AD/HS, AC/HS; **Tables S7A, C, E, G, I, K**). The 483 genes upregulated in ACLF/HS were significantly enriched in genes involved in innate immunity, including neutrophil, monocyte, Toll-like receptor and inflammatory signaling genes (**Figure 2D**, left) (**Table S7A**). In addition, analysis of Reactome pathways identified enrichment in neutrophil granule genes (**Table S7C**). Importantly, the lists of upregulated genes in the two other comparisons (AD/HS, AC/HS) had a totally different enrichment profile (**Figure 2D**, left; **Tables S7E, G, I, K**), indicating the specificity of the innate inflammatory gene signature in blood from patients with ACLF.

To capture the dynamics of upregulation of co-expressed genes across study groups, we computed gene clusters using whole-blood transcriptomic data, with the transcriptome in HS as a reference (Supplementary Methods). Each cluster was composed of significantly correlated expression values across the three patients’ groups; a gene included in a given cluster was not present in another cluster. We identified nine co-expression gene clusters (**Figure S6A**, **Table S8A**), which were numbered arbitrarily by the algorithm, and included a total of 2116 genes. For each cluster, we computed an eigengene (21) which is a gene summarizing the global behavior of the cluster member genes (**Figures S6A, B**). Cluster 2 was interesting because it was the second largest cluster (416 genes) and 70% of the cluster member genes were upregulated in ACLF/HS. Cluster 2 eigengene was upregulated in ACLF versus the other patients’ groups and did not differ between AC and AD, indicating that cluster 2 captured gene upregulation that was specific for ACLF (**Figure S6B**). Finally, the profile of enrichment of cluster 2 (**Figure S6C**, **Table S8**) was very similar to that of upregulated genes in ACLF/HS, in particular member genes were enriched in innate inflammatory genes. Together these findings confirmed that the orchestrated activation of the blood innate inflammatory signature was a molecular hallmark of ACLF.

**Figure 3:**
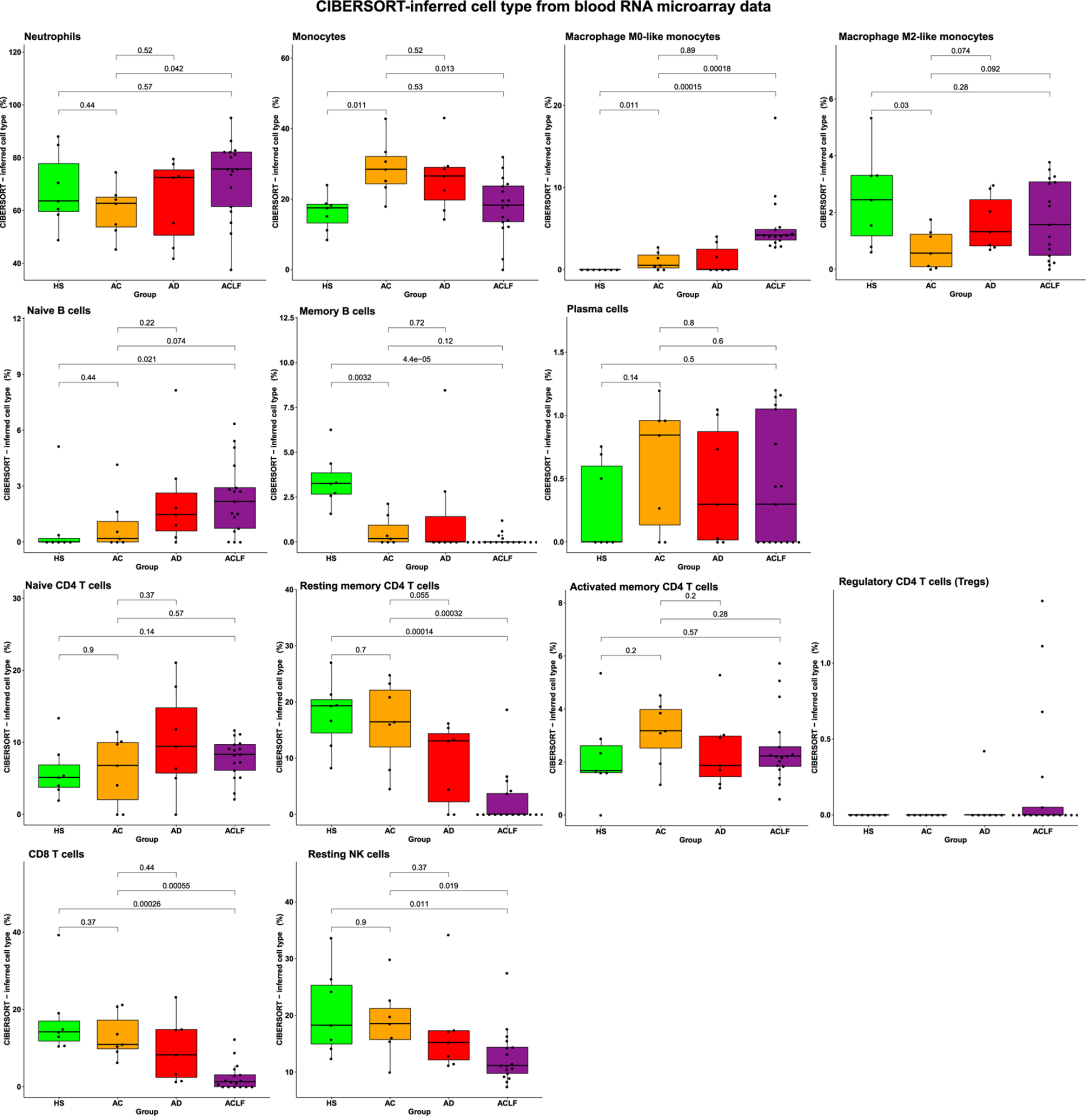
The CIBERSORT method identifies alterations in blood immune-cell subset composition in ACLF. There were seven healthy subjects (HS), seven patients with advanced cirrhosis (AC), seven patients with acutely decompensated cirrhosis (AD), and 17 patients with ACLF. P values were obtained using one-way ANOVA and unpaired *t*-tests.

One additional mean of deconvoluting the RNA microarray data, the CIBERSORT software package (22), showed that there were no significant differences in the proportions of myeloid cells between AC and AD, and that these two groups had modest changes restricted to the blood monocyte/macrophage compartment relative to HS (**Figure 3**). In contrast, blood in ACLF had significant increases in the proportions of neutrophils (relative to AC) and macrophage M0-like monocytes (relative to HS and AC) (**Figure 3**). Using flux cytometry in additional individuals, we found that ACLF was associated with a significant increase in the frequency of a monocyte subset (**Figure S7**). Together these findings indicate that, in blood from patients with ACLF, the development of an innate inflammatory gene signature coincided with increases in certain myeloid cell populations (neutrophils and macrophage M0-like monocytes) that were unique to these patients.

#### ACLF Associates With Depletion of Several Blood Lymphocyte Subsets

Using the fold-changes values for the 50 most downregulated genes in ACLF/HS, and the corresponding values in the two other comparisons (**Table S6**), we drew a Cleveland plot showing fold-changes for each gene across the three pairwise comparisons (**Figure 2C**, bottom). For each gene, the fold-change was greater in ACLF/HS than the fold-change in the two other comparisons. However, in both AC/HS and AD/HS, several genes fulfilled the criteria defining downregulation (**Figure 2C**, bottom; **Table S6**), suggesting a progressive decrease in the expression of these genes from AC or AD to ACLF. For example, *TCF7* and *LEF1*, that are crucial for establishment of central memory CD8 T cells, were downregulated, respectively, by 1.6-, 1.6-, 2.7-fold and by 1.6-, 1.8-, 4.3-fold, in AC/HS, AD/HS, and ACLF/HS (**Table S6**).

Then, we performed BTM-based analyses and found that the 1,086 downregulated genes in ACLF/HS were significantly enriched in T-cell and NK-cell genes (**Figure 2D**, right; **Table S7B**). Moreover, Reactome pathways analysis showed that downregulated genes in this comparison were significantly enriched in genes related to RNA metabolism and translation of RNA into proteins (**Table S7D**), indicating a decrease in the constitutive apparatus required for gene induction and expression, consistent with an increased threshold for lymphocyte activation. Importantly, the lists of downregulated genes in the two other pairwise comparisons (AC/HS, AD/HS) were also significantly enriched in T-cell, and NK-cell genes (**Figure 2D**, right; **Tables S7F, J**), and genes related to RNA metabolism and translation (**Tables S7H, L**) confirming that lymphocyte gene downregulation was not unique to ACLF. Of note, our “home-made” analysis revealed that the eigengene representative of the largest gene cluster (cluster 3, 995 genes) progressively decreased from HS to ACLF; the decrease was already significant in AC relative to HS and reached its maximum with ACLF (**Figure S6B**). Ninety-two percent of cluster 3 member genes were downregulated in ACLF/HS. Moreover, the profile of enrichment of cluster 3 (**Table S8**) was very similar to that of downregulated genes in ACLF/HS, in particular member genes were enriched in genes related to T cells, B cells, and NK cells. Together these findings confirmed that decompensated cirrhosis was characterized by a progressive decline in the expression of genes related to lymphocytes, the lowest gene expression being observed in ACLF.

The use of the CIBERSORT method showed that ACLF was associated with significant decreases in several lymphocyte populations including memory B cells; resting memory CD4 T cells; CD8 T cells; and NK cells (**Figure 3**). The ACLF-associated depletion was restricted to these cells because the proportions of plasma cells; naïve CD4 T cells; activated memory CD4 T cells; and regulatory CD4 T cells (Tregs) were not affected in ACLF (**Figure 3**). Of note, there were no significant alterations in lymphocyte proportions in AC and AD (**Figure 3**). Together, these results indicated that the depletion of certain blood lymphocyte subsets was unique to ACLF, and contributed to both the decrease in clinical differential lymphocyte counts (**Figure 1A**) and the maximum reduction in lymphocyte-related genes (**Figure 2D**), observed in this syndrome. In addition, the ACLF-associated lymphocyte depletion measured with the CIBERSORT method was consistent with results of our analysis of the blood lymphocyte compartment using flux cytometry in additional individuals (**Figure S8**). In addition, flux cytometry identified a significant decrease in NK T cells (which are innate-like killer T cells; **Figure S8**).

### Unique Phenotype of Circulating Neutrophils in ACLF

#### Blood Neutrophils Are Activated in ACLF

**Figure 4:**
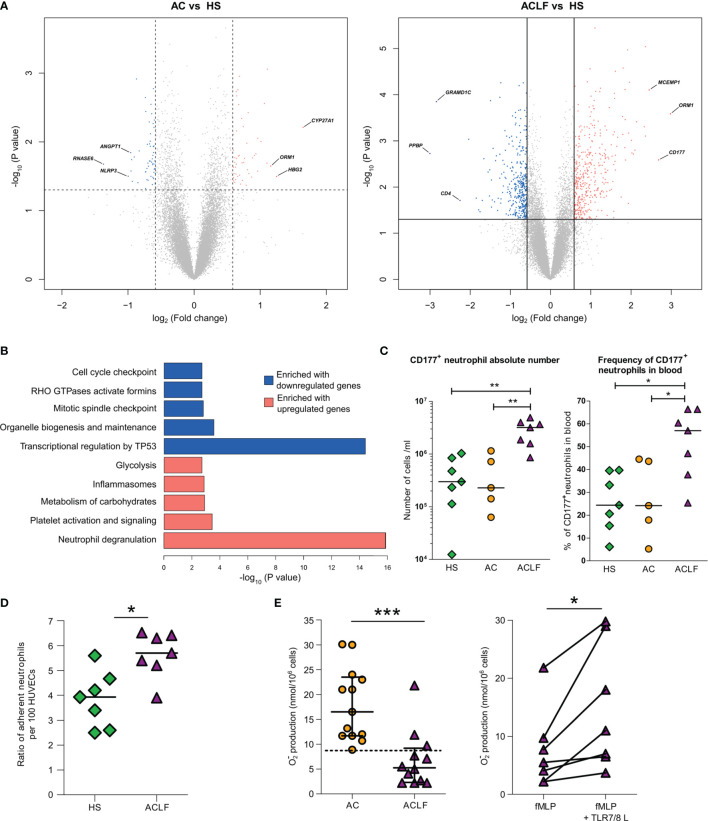
Unique phenotype of neutrophils from patients with ACLF. **(A)** Volcano plots of differential neutrophil gene expression between AC and HS and ACLF versus HS. There were five healthy subjects (HS), five patients with advanced cirrhosis (AC), and five patients with ACLF. Between-group comparisons were performed using unpaired Student’s *t*-test. *CD177* was the most upregulated gene in ACLF versus HS. **(B)** Enrichment of Reactome data sets with differentially expressed genes between ACLF and HS. **(C)** Absolute number and frequency of CD177^+^ neutrophils in blood from HS (green diamonds, n=7), patients with AC (orange circles, n=5) and patients with ACLF (purple triangles, n=7). **(D)** Increased adhesion to HUVECs of neutrophils from ACLF patients relative to neutrophils from HS. Neutrophils from HS (n=7) and patients (n=7) were freshly isolated; their adherence to HUVECs was measured using flow cytometry as the ratio of CD66b^+^ neutrophil per HUVEC. **(E)** Defective superoxide production in neutrophils from patients with ACLF is restored by a TLR7/8 agonist. Freshly isolated neutrophils from 13 patients with ACLF and 13 patients with AC were stimulated with fMLP (1 µM) and respiratory burst was monitored using the cytochrome c reduction assay. In seven patients, neutrophils were pre-treated with the TLR7/8 agonist CL097 (2 µg/ml) for 10 min before stimulation with fMLP. During the same period, median [IQR] superoxide production in neutrophils from healthy subjects was 19.6 [15.4–27.25]. ***P <0.001; *P <0.05. HUVEC denotes human umbilical vein endothelial cell, and fMLP N-Formylmethionyl-leucyl-phenylalanine.

Because increased blood neutrophil population was a major feature of ACLF (**Figure 1A**), we performed genomewide analysis of RNA expression in freshly isolated peripheral-blood neutrophils from 15 additional individuals (five HS, five patients with AC, and five patients with ACLF; **Tables S1** and **S9**). There were only 140 DEGs (71 upregulated) in AC/HS and 832 DEGs (420 upregulated) in ACLF/HS (**Figure 4A**; **Table S10A**). Next, we focused on the list of DEGs in ACLF/HS, and pathway analysis of upregulated genes identified that 48 of these genes were neutrophil granule genes (**Figure 4B**; **Table S10B**), findings consistent with analysis of upregulated genes in whole-blood (**Figure 2D**). Granule genes encode proteins which are expressed in the lumen or membrane of different types of neutrophil granules under steady-state, and are released *via* exocytosis, following neutrophil activation (25). *CD177*, which was the second most upregulated gene in ACLF/HS (**Figure 4A**), was in the list of 48 upregulated granule genes. The CD177 protein resides in the granule membrane under basal conditions, and is mobilized to the cell surface following neutrophil activation (25, 26). Using flow cytometry, we investigated CD177 protein expression in neutrophils obtained from two additional series of individuals, one enrolled in France (seven HS, five patients with AC, and seven with ACLF, **Table S1**) and the other in India (eight patients with AC and 12 with ACLF; **Tables S1** and **S11**). The results obtained in both series showed that patients with ACLF had increases in the absolute number and frequency of CD177^+^ neutrophils (**Figure 4C** and **Figure S9**, for French and Indian patients, respectively). Moreover, mean fluorescence intensity was significantly increased in neutrophils from French patients with ACLF (**Figure S10**), confirming increased CD177 expression in these cells. Of note, upregulated genes in ACLF/HS were significantly enriched in genes related to carbohydrate metabolism (**Figure 4B**), consistent with metabolic reprogramming in activated neutrophils (27). Collectively, our analysis of upregulated genes in ACLF/HS indicated that circulating neutrophils were activated in ACLF. In addition, pathway analysis of downregulated genes in ACLF/HS revealed that these genes were related to TP53-induced transcription (involved in cell death), activation of formins (involved in cytokinesis), cell cycle, and mitosis (both involved in proliferation) (**Figure 4B**; **Table S10C**), which are other features of unique neutrophil phenotype in ACLF (28).

#### Blood Neutrophils From Patients With ACLF Have Increased Adhesion to Endothelial Cells

Expression of the CD177 protein at the neutrophil plasma membrane is known to result in neutrophil arrest at the endothelium surface (26). Because of the elevated frequency of CD177^+^ cells among neutrophils from patients with ACLF, we hypothesized that these neutrophils would exhibit enhanced adhesion to endothelial cells as compared with neutrophils from HS. We freshly isolated neutrophils from patients with ACLF and HS and compared, *in vitro*, their adhesion to human umbilical vein epithelial cells (HUVECs) (29). Patients’ neutrophils had an enhanced adherence to HUVECs (**Figure 4D**). Whether this effect directly or indirectly dependent on CD177 overexpression should be further investigated.

#### In ACLF, Blood Neutrophils Have Defect in Superoxide Anion Production That Is Pharmacologically Reversible

Neutrophils are the first-line defense against bacterial infections (30). When stimulated by bacterial cues, such as N-Formylmethionyl-leucyl-phenylalanine (fMLP), neutrophil NADPH oxidase is activated to produce large amount of the antimicrobial superoxide anion (respiratory burst) (30). In its active form, NADPH is a multiprotein complex comprising NOX2 which catalyzes superoxide anion production (30). We (15, 16), and others (14), have shown that compared with neutrophils form HS, neutrophils from patients with AC have a defective of superoxide anion production in response to fMLP. This defect is explained, at least in part, by a degradation of the protein NOX2 involving the granule protein elastase that has been released *via* degranulation (15). Because, as mentioned earlier, there was evidence of marked neutrophil degranulation in ACLF, we wondered whether superoxide anion production was affected in neutrophils from patients with ACLF. To address this question, neutrophils from two groups of patients (ACLF and AC) were isolated to investigate the production of superoxide anion in response to the bacterial fMLP (Supplementary Methods, **Table S1**). FMLP-induced superoxide anion production by neutrophils was significantly lower in patients with ACLF than in those with AC (**Figure 4E**), suggesting that ACLF was associated with a major defect in NADPH oxidase activation.

We have previously shown that neutrophils isolated from patients with cirrhosis and exposed to CL097 or R848 (which are agonists for endosomal TLR7/8) had an increased superoxide production in response to fMLP (15). We investigated the effects of CL097 on the response to fMLP in neutrophils from patients with ACLF and found that CL097 enhanced fMLP-induced superoxide production in these cells (**Figure 4E**). These findings suggest that the mechanisms resulting in defective superoxide production in fMLP-stimulated neutrophils from patients with ACLF were reversible with endosomal TLR7/8 engagement.

## Discussion

This pilot study is the first to investigate the landscape of circulating immune cells in critically ill patients with ACLF seen during the first 24 h of their ICU stay. We used results of clinical complete blood count measurements and microarray (genomewide) analysis of blood RNA expression, in HS and 3 groups of patients with cirrhosis, comprising AC, AD, and ACLF. In addition to traditional bioinformatic analysis of transcriptomic data, deconvolution of these data was performed using the CIBERSORT method, enabling to enumerate the proportions of immune-cell subsets present in a given tissue, here the blood (22). The key results were that patients with ACLF had dysregulation of certain circulating immune cells, including leukocytosis fueled by increased populations of neutrophils (that had unique phenotype) and macrophages M0-like monocytes, and, as expected (31, 32), decreases in lymphocyte count related to a depletion in memory lymphocytes (of the B-cell, CD4 T-cell lineages), CD8 T cells and NK cells. Flux cytometry also identified that ACLF was associated with a decrease in circulating NK T cell population. The dysregulation of blood immune cells was unique to ACLF because it was not observed in AC and AD.

One cannot exclude that increases in blood neutrophil population associated with ACLF was partly due to the mobilization of a marginated pool of neutrophils (26). However, a more likely explanation for neutrophilia is the stimulation of a hematopoietic response program called emergency granulopoiesis (28, 33). Under acute inflammatory stresses, such as sepsis, PAMPs, cytokines (granulocyte colony-stimulating factor [G-CSF], IL-1, TNF-α), or both, stimulate large-scale *de novo* production of neutrophils from myeloid precursors in the bone marrow (33). Patients with ACLF have increased systemic levels of stimuli for emergency granulopoiesis, including PAMPs (lipopolysaccharide) (2) and cytokines, including G-CSF, IL-1, TNF-α (2–4) (**Table S12**).

In this study, microarray analysis of blood neutrophil RNA expression enabled us to show that neutrophils from patients with ACLF overexpressed genes encoding granule proteins (**Table S9**), including *CD177*, which was among the most upregulated transcript in these cells. Flow cytometry revealed that the frequency of CD177 was higher in neutrophils from patients with ACLF. CD177 degranulation at the neutrophil plasma membrane is a signal for neutrophil arrest at the endothelium surface in tissue vessels (26). Thus, using *ex vivo* experiments of neutrophil adhesion to HUVECs, we found that neutrophils from patients with ACLF were more adherent to endothelial cells than their counterpart from HS. The interaction of activated neutrophils with endothelial cells may cause endothelium injury and activate local prothrombotic mechanisms (34). Consistent with this hypothesis, patients with ACLF had elevated plasma levels of markers for endothelial dysfunction (**Table S12**). Therefore, an increased frequency of CD177^+^ neutrophils in ACLF might play a major role in the development of organ failures in this syndrome. This could also be the case in patients without cirrhosis who have septic shock because previous studies have shown that CD177 was most upregulated gene and protein in neutrophils from these patients (35).

The ACLF-associated depletion in circulating lymphocytes can have different explanations which are not mutually exclusive. First, like patients of the general population who have protracted sepsis, patients with ACLF may have excessive blood lymphocyte death (36). Second, one cannot exclude another that, like in other severe acute inflammatory diseases (37), ACLF was associated with an increased egress of lymphocytes, from blood to lymphoid or non-lymphoid tissues, that would contribute to circulating lymphopenia. Of note, however, in patients without cirrhosis who had protracted sepsis, blood lymphopenia was associated with extensive loss of lymphocytes in spleens, intestines, and other organs (38). Future studies are needed to elucidate the mechanisms of blood lymphopenia in blood from patients with ACLF.

Patients with ACLF have immunosuppression which is indicated by the high incidence of new or secondary bacterial of fungal infections in these patients (12, 13). Mechanisms driving immunosuppression in ACLF may include increased numbers of MerTK-expressing monocytes (4), and myeloid-derived suppressor cells (9), and exhausted T cells (39). Moreover, our study suggests additional mechanisms for immunosuppression in ACLF, related to alterations in both neutrophils and the lymphoid lineage. Indeed, although neutrophils from patients with ACLF had features of activation, these cells were defective in fMLP-induced neutrophil production of the antimicrobial superoxide anion (**Figure 4E**). This defect, therefore, was a factor of ACLF-associated systemic immunosuppression. Using the CIBERSORT method, we also found that ACLF was associated with a systemic depletion of resting memory CD4 T cells which may play a role in systemic immunosuppression in this syndrome; thus, memory CD4 T cells are essential for an immediate response against bacterial infections (40). Moreover, CD8 T cells, which are involved in elimination of infected cells (41), had a decreased proportion in ACLF. In addition, the populations of NK cells and NK T cells, which play an important role in the innate immune response against microbes (including fungi) (42) were significantly decreased in blood from patients with ACLF. Of note, the CIBERSORT method did not provide explicit information on CD8 T cells states such as exhaustion and memory. Because increased exhausted CD8 T cells and decreased in memory CD8 T cells are important contributors for immunosuppression (34, 43), we established a list of genes that are markers for each of these states (**Table S13**). In our patients with ACLF, we did not find evidence of an induction of exhaustion markers (such as negative checkpoint regulators), but identified a downregulation of the gene signature of memory CD8 T cells, suggesting that these cells may be involved in immunosuppression associated with ACLF.

A decreased number of memory CD8 T cells are involved in immunosuppression associated with cancer (43) or sepsis (36). In the context of cancer, generating more memory CD8 T cells (for example, through IL-7 or IL-15 pathways) may improve the outcomes (43). Similar approaches are considered as being of potential interest for treating immunosuppression in patients with sepsis (36). Our finding, in ACLF, of downregulation of the gene signature of memory CD8 T cells may suggests that immunotherapies with IL-7 or IL-15 might be of interest against immunosuppression associated with this syndrome. Of note, the blood expression of *IL2RG*, encoding the γ-chain receptor which is shared by IL-7 and IL-15 and is indispensable for signaling of these cytokines in target cells, was not affected in ACLF (**Table S6**). Finally, our result, that a TLR7/8 agonist stimulated production of superoxide anion in neutrophil from patients with ACLF, requires confirmation because it suggests another approach against immunosuppression associated with ACLF.

Our study has limitations, in particular because of the small number of patients investigated. However, our study enabled to validate deconvolution of RNA microarray data as a means to enumerate immune-cell subsets in the blood of patients with cirrhosis. This approach may therefore be used in large longitudinal studies assessing, for example, the impact of therapies on systemic inflammatory cells in patients with ACLF. It is also important to note that, although a co-orchestrated decrease in a large number of lymphocyte genes were significantly downregulated in AD and AC relative to HS (**Figure 2D**; **Figure S5**), the CIBERSORT-inferred proportions of lymphocyte subsets did not significantly differ in AD/HS and AC/HS (**Figure 3**). These findings, therefore, were in sharp contrast with those obtained in ACLF/HS where maximum lymphocyte gene downregulation (**Figure 2D**) coincided with decreases in certain blood lymphocyte populations (**Figure 3**). Collectively, our results suggested a model, in which blood lymphocyte gene downregulation was a characteristic of decompensated cirrhosis that preceded the decrease in the frequency of certain circulating lymphocytes; this decreased frequency being the hallmark of ACLF.

In conclusion, this pilot study showed that patients with ACLF had dysregulation in systemic immune cells including leukocytosis fueled by neutrophils and a monocyte/macrophage subset, and decreases in lymphocyte count related to a depletion in memory lymphocytes, CD8 T cells, and NK cells. All these lymphocyte alterations, along with blood enrichment in neutrophils defective in superoxide anion production, may contribute to immunosuppression in ACLF. Finally, our study suggests the existence of targets for novel therapeutic approaches for decreasing the risk of infection among patients with ACLF.

## Data Availability Statement

The datasets presented in this study can be found in online repositories. The names of the repositories and accession numbers can be found here: https://www.ncbi.nlm.nih.gov/geo/, GSE142255 (whole-blood samples); https://www.ncbi.nlm.nih.gov/geo/, GSE142254 (neutrophil samples).

## Ethics Statement

The studies involving human participants were reviewed and approved by Comité de protection des personnes Ile de France III N°3194 and Comité d’Evaluation de l’Ethique des projets de Recherche Biomédicale (CEERB) Paris Nord (IRB 00006477 n°13-043 in France F.25/5/81/ILBS/AC/2015/910 in India). The patients/participants provided their written informed consent to participate in this study.

## Author Contributions

Study concept and design (RM, EW, PG). Acquisition of transcriptomic data (PG, AJ). Acquisition of clinical data and samples (EW, MD, LM, SS, BA, HG, MT, GM, CF, P-ER). Bioinformatic and statistical analyses (PG, JL, FA, AJ, EW, RM). Acquisition of *ex vivo* data (PH, LM, MT, JP, SS, JC). Integration of clinical and biological results and interpretation of data (PG, JL, FA, AJ, P-ER, EW, RM). Drafting of the manuscript (RM, EW, PG). Critical revision of the manuscript for important intellectual content (JC, AP, GM, RJ, CF, P-ER, SL, VA, FD). Study supervision (RM). All authors contributed to the article and approved the submitted version.

## Funding

The study was supported by INSERM, the Fondation pour la Recherche Médicale (FRM grant number DEQ20150331726 to SL), and the European Foundation for the Study of Chronic Liver Failure (EF-Clif). The EF Clif is a non-profit private organization.

## Conflict of Interest

PG and AJ were employed by GenoSplice. RJ has research collaborations with Yaqrit and Takeda. RJ is the inventor of OPA, which has been patented by UCL and licensed to Mallinckrodt Pharma. He is also the founder of Yaqrit limited, a spin out company from University College London and Thoeris Ltd. FD consults and has received grants from Gilead and Astellas.

The remaining authors declare that the research was conducted in the absence of any commercial or financial relationships that could be construed as a potential conflict of interest.

## Abbreviations

ACLF, acute-on-chronic liver failure; CIBERSORT, cell-type identification by estimating relative subsets of RNA transcripts; AD, acute decompensation; AC, advanced cirrhosis; HUVEC, human umbilical vein epithelial cell; fMLP, N-Formylmethionyl-leucyl-phenylalanine.
