# Supplementary material for: Characterization of Blood Immune Cells in Patients With Decompensated Cirrhosis Including ACLF
Source: Front Immunol. 2021 Feb 5;11:619039. doi: 10.3389/fimmu.2020.619039 (PMC7893087; doi:10.3389/fimmu.2020.619039)
Supplement: Supplementary file 1 [file DataSheet_1.zip › Supplementary material/Supplementary_Appendix_Frontiers_R1.doc]

**Manuscript ID: 619039_R1_draft 2**

**Supporting Information**

This appendix has been provided by the authors to give readers additional information about their work entitled “RNA Identification of Dysregulated Blood Immune Cells Playing a Pathophysiological Role in Patients With ACLF**”**

Supplement to:

Emmanuel Weiss,^1,2,3,†^ Pierre de la Grange,^4,†^ Mylène Defaye,^2^ Juan José Lozano,^5^ Ferran Aguilar,^3^ Pushpa Hedge,^2^ Ariane Jolly,^4^ Lucile Moga,^2,6^ Sukriti Sukriti,^7^ Banwari Agarwal,^8^ Haqeeqat Gurm,^8^ Marion Tanguy,^2^ Johanne Poisson,^2^ Joan Clària,^3,5,9,10^ Paer-Selim Abback,^1,2^ Axel Périanin,^2^ Gautam Mehta,^8,11^ Rajiv Jalan,^3,8^ Claire Francoz,^6^ Pierre-Emmanuel Rautou,^2,6^ Sophie Lotersztajn,^2^ Vicente Arroyo,^3^ François Durand^2,6,††^, Richard Moreau^2,3,6,††^

^†^Share first authorship.

^††^Co-senior authors.

^1^Assistance Publique – Hôpitaux de Paris (AP-HP), Department of Anesthesiology and Critical Care, Beaujon hospital, DMU Parabol, AP-HP Nord, Paris;

^2^ Université de Paris, Institut National de la Santé et de la Recherche Médicale (INSERM), Centre de Recherche sur l’Inflammation (CRI), Paris, France;

^3^EF Clif, EASL-CLIF Consortium and Grifols Chair, Barcelona, Spain;

^4^Genosplice, Paris, France;

^5^CIBERehd, Barcelona, Spain;

^6^Assistance Publique – Hôpitaux de Paris (APHP), Service d’Hépatologie & Réanimation HépatoDigestive, Hôpital Beaujon, Clichy, France

^7^Department of Research Institute of Liver and Biliary Sciences, New Delhi, India;

^8^Liver Failure Group, Institute for Liver and Digestive Health, University College London, Royal Free Campus, London, United Kingdom;

^9^Hospital Clínic-IDIBAPS, Barcelona, Spain;

^10^Universitat de Barcelona, Barcelona, Spain;

^11^Institute of Hepatology, Foundation for Liver Research, London, United Kingdom; Faculty of Life Sciences & Medicine, King's College London, London, United Kingdom

**Supplementary Appendix**

Table of contents

| **Category** | **Title** | **Page** |
| --- | --- | --- |
| Text | Patients and methods. | 6-13 |
| Table S1 | Study protocols according to the characteristics and origin of the enrolled participants. | 14-15 |
| Table S2 | Definitions of the CLIF Consortium for organ system failures, acutely decompensated cirrhosis, and acute-on-chronic liver failure (ACLF) used in the present study. | 16-17 |
| Table S3 | List of the 349 blood transcription modules (BTMs) and their member genes used as gene sets for enrichment analysis. | Separate Excel file |
| Table S4 | Characteristics of the English patients with acutely decompensated cirrhosis with and without ACLF. | 18-20 |
| Table S5 | Individual results of the CIBERSORT method that were used to calculate the CIBERSORT-inferred blood counts. | Separate Excel file |
| Table S6 | Lists of genes that were differentially expressed or not in ACLF versus HS, ranked according to fold-changes in gene expression, from the highest to the lowest fold-change value. The fold changes of each gene calculated for the 2 other pairwise comparisons (AC/HS, AD/HD) were also given. The Table S6 was prepared to obtain the Cleveland plot shown in Fig. 2C. This Table also shows gene differentially expressed in ACLF versus AC. | Separate Excel file |
| Table S7 | Results of enrichment analysis for 3 lists of DEGs (ACLF/HS, AD/HS, AC/HS). | Separate Excel file |
| Table S8. | List of member genes of each of the 9 co expression clusters and their enrichment in the BTM space and Reactome gene sets. | Separate Excel file |
| Table S9 | Characteristics of the French patients with advanced cirrhosis (AC) and those with ACLF in whom neutrophil transcriptome was investigated. | 21-24 |
| Table S10 | List of differentially expressed neutrophil genes in 2 pairwise comparisons (advanced cirrhosis [AC] vs. healthy subjects [HS]; ACLF vs. HS), and results of enrichment analysis of DEGs in ACLF vs. HS, using predefined Reactome gene sets. | Separate Excel file |
| Table S11 | Characteristics of Indian patients with advanced cirrhosis (AC) and with ACLF. | 25-27 |
| Table S12 | Plasma levels of cytokines, chemokines, growth factors, markers of macrophage activation, coagulation/platelet function and endothelial dysfunction in healthy subjects and patients with ACLF, all enrolled in France. | 28-31 |
| Table S13 | Bone fide genes related to memory and exhausted CD8 T cell states, which are present or not among in the significantly differentially expressed genes between ACLF and HS, using whole-blood transcriptomic data (Table S5). | 32-33 |
|  |  |  |
| Fig. S1 | Absolute counts of neutrophils, monocytes, and lymphocytes in AC, AD, and ACLF | 34 |
| Fig. S2 | Complete clinical blood count measurements in the English cohort of patients with acutely decompensated cirrhosis showed that ACLF associated with leukocytosis, neutrophilia, and lymphopenia. | 35 |
| Fig. S3 | Unsupervised hierarchical clustering of whole-transcriptome measured in the 4 study groups of French patients, including patients with advanced cirrhosis (AC), patients with acute decompensation of cirrhosis (AD), patients with ACLF, and healthy subjects (HS). | 36-37 |
| Fig. S4 | Results of RT-qPCR measurements in a random set of genes using RNA extracted for whole-blood transcriptomics in French patients with advanced cirrhosis (AC) and those with ACLF. | 38-41 |
| Fig. S5 | Volcano plot of differential gene expression between patients with ACLF (n=17) relative to healthy subjects (n=7). | 42 |
| Fig. S6 | Nine gene clusters capture the dynamics of gene co-expression across all study groups. | 43-44 |
| Fig. S7 | Analysis by flow cytometry of circulating monocyte subsets in healthy subjects (HS), patients with advanced cirrhosis (AC), and patients with ACLF. | 45 |
| Fig. S8 | Analysis by flow cytometry of circulating subsets of T cells, B cells and natural killer (NK) T cells in healthy subjects, patients with advanced cirrhosis (AC), and patients with ACLF. | 46 |
| Fig. S9 | CD177^+^ neutrophils in blood from Indian patients with advanced (AC) cirrhosis and patients with ACLF. | 47 |
| Fig. S10 | Mean Florescence Intensity (MFI) of CD177 in blood neutrophils from French individuals including healthy subjects (HS), patients with advanced cirrhosis (AC), and patients with ACLF. | 48 |
|  |  |  |
| References |  | 49-50 |

**Supplementary Methods**

**Patients**

The study protocol was approved by the ethics committees (Comité de protection des personnes Ile de France III N°3194 and Comité d’Evaluation de l’Ethique des projets de Recherche Biomédicale [CERB] Paris Nord [IRB 00006477 n°13-043]) in France; and F.25/5/81/ILBS/AC/2015/910 in India. Informed consent was obtained for all patients.

In France, 99 patients with cirrhosis were enrolled at Beaujon Hospital (Assistance Publique - Hôpitaux de Paris, Clichy). Among these, 64 were nonelectively admitted to the hospital, including 57 patients with critically ill ACLF (admitted to the Hepatology and Liver Intensive Care [ICU]), and 7 patients with acute decompensation of cirrhosis without ACLF (this group being called hereafter “AD group”). The remaining 35 patients had stable decompensated cirrhosis (this group being hereafter called advanced cirrhosis [AC]), were enrolled when electively admitted to the regular ward of the liver unit. Of note, patients with AC did not have any ongoing or recently treated (less than 1 week) bacterial infection or gastrointestinal hemorrhage. In England, 203 patients with ACLF and 91 with AD were enrolled in the ICU of University College London (London). Finally, in India, 12 patients with ACLF and 8 with AD were enrolled in the ICU at the Institute for Liver and Biliary Sciences (New Delhi).

In France, blood from 33 healthy volunteers was obtained by Etablissement Français du Sang (agreement n° 2015012778).

Assignment of patients to different protocols are shown in Table S1.

**Definitions**

Organ system failures were defined according to the Chronic Liver Failure Consortium (CLIF) Organ Failure scoring system (Table S2).

**Methods**

*Microarray (genomewide) analysis of RNA expression in blood*

Blood from 17 patients with ACLF, 7 patients with AD cirrhosis and 7 patients with AC was collected in Tempus tubes (Thermo Fisher Scientific, Waltham, MA, USA). RNA was extracted using the 5 PRIME PerfectPure RNA blood kit (Thermo Fisher Scientific) according to manufacturer’s instructions. One hundred nanograms of RNA per sample were then hybridized on Human Transcriptome Array 2.0 (HTA2.0, Affymetrix, Santa Clara, CA) at the genomic platform of the Curie Institute (Paris, France). This array was designed to measure the RNA expression of 67,528 genes.

**Whole-blood microarray data analysis.** HTA2.0 dataset analysis and visualization were made using EASANA® (GenoSplice technology), which is based on the GenoSplice’s FAST DB® annotations.[1,2] HTA2.0 data were normalized using quantile normalization. Background corrections were made with antigenomic probes and probes were selected as described previously.[3] Only probes targeting exons annotated from FAST DB® transcripts were selected to focus on well-annotated genes whose mRNA sequences are in public databases.[4,5] Bad-quality selected probes (e.g., probes labeled by Affymetrix as ‘cross-hybridizing’) and probes whose intensity signal was too low compared to antigenomic background probes with the same GC content were removed from the analysis. Only probes with a DABG P value ≤0.05 in at least half of the arrays were considered for statistical analysis.[3] Only genes expressed in at least one compared condition were analyzed. To be considered as expressed, the DABG P value had to be <0.05 for at least half of the gene probes. We performed a Student’s *t*-test to compare gene intensities in the different biological replicates. Genes were considered significantly regulated when fold-change was >1.5 and P value <0.05.

**Construction of gene clusters and identification of eigengenes summarizing their behavior.** To analyze the dynamics of whole-blood transcription across our 4 study groups, we used whole-blood transcriptomic data to identify clusters that were composed of genes co-expressed across these groups (Pearson’s correlation coefficient ≥ 0.5) with limited variation across samples from the same group (variance ≤2.0). Of note, a given gene was member of a single cluster gene and only clusters with more than 50 member genes were considered. For each cluster, an eigengene summarizing its behavior was computed (Langfelder and Horvath).

Biological interpretation of our gene clusters was performed using the 346 blood transcription modules (BTM) previously developed by Li, Rouphael, Duraisingham et al [6]. Briefly, each BTM was contained genes related to an immune cell subset, a molecular function, a signaling pathway or a biological process, that were co-expressed in peripheral-blood mononuclear cells from individuals under various immunological conditions. In our work, the BTMs were used as gene set for enrichment analysis. In addition, we performed enrichment analysis of cluster genes using publicly available gene sets provided by Reactome (Reactome.org).

**CIBERSORT deconvolution of RNA microarray data.** We applied CIBERSORT,[7] using a signature matrix (LM22) file consisting of 547 genes that accurately distinguish 22 mature human hematopoietic populations isolated from peripheral-blood mononuclear cells, for deconvolution of our dataset and to estimate the absolute proportions of these cell types in our whole-blood samples. Analyses were done with 1000 permutations, disabled quantile normalization and default statistical parameters. The results were filtered by a maximum P value <0.05.

*Reverse transcription quantitative polymerase chain reaction (RT-qPCR)*

A random set of genes was selected to compare results obtained with microarrays and those obtained using RT-qPCR. As previously described, total RNA (1 µg) was reverse transcribed with Transcriptor cDNA Synthesis kit (Roche Diagnostics, Basel, Switzerland).[8] Syber Green PCR master mix and a LightCycler 4800 real-time thermocycler (Roche Diagnostics) were used for quantitative PCR and assays were performed in duplicate. A melting curve was systematically run at the end the reaction to verify that the used primer pair produced a single product. Furthermore, to favor technical reproducibility of the RT-qPCR measurements, samples with standard deviation at the crossing point (CP) >0.3 from the mean CP were excluded from analysis. Percent PCR amplification efficiencies (E) for each assay were calculated as E = (10-1/slope - 1) × 100, using the slope of the semi-log regression plot of Ct versus log input of cDNA (10-fold dilution series of five points). A threshold of 5% above or below 100% efficiency was applied (i.e. an efficiency between 1.9 and 2.1). Then, results were normalized to those of the GAPDH housekeeping gene and expressed as fold change relative to un-stimulated value using the following formula: 2^-∆∆Ct^ (2^-∆∆Ct^ values of unstimulated samples were arbitrarily set at 1).

*Flow cytometry analysis of blood immune cell subsets*

One milliliter of whole blood was centrifuged (10 min, 200g) and resuspended in red blood cell lysis buffer (Biolegend, San Diego, CA). After 15 minutes, cells were washed twice with FACS buffer (1xPBS with 0.1% FBS). The cells were stained with fluorochrome conjugated anti-human CD3 (OKT3), CD4 (OKT4), CD14 (HCD14), CD15 (W6D3), CD16 (BV510), CD19 (H1B19), CD33 (P67.6), CD56 (5.1H11) and CD177 (MEM-166) antibodies obtained from BioLegend, France). Anti-CD8 (SK1) was obtained from BD Biosciences. Immune subsets were identified by forward and side scatter and combinations of the following markers: monocytes (CD14^+^/CD16^+^), T cells (CD3^+^/CD4^+^ or CD3^+^/CD8^+^), NKT cells (CD3^+^/CD56^+^), B cells (CD19^+^) and neutrophils (CD15^+^/CD16^+^/CD33^+^). The samples were acquired using BD FACS Fortessa (French cohort) or BD FACSverse (Indian cohort) and analyzed using FlowJo software (BD Biosciences, San Jose, CA).

*Microarray analysis of RNA expression in blood neutrophils*

**Neutrophil isolation.** Neutrophils were isolated as previously described.[9] Briefly, neutrophils were isolated from peripheral blood (collected in EDTA tubes) obtained in 25 patients with ACLF, 23 patients with AC, and 12 healthy donors. Whole blood was diluted with PBS (1:1) and mononuclear cells were isolated from neutrophils and erythrocytes after centrifugation on a cushion of Histopaque 1077™ (Sigma, Saint Louis, MO) gradient at (400 g for 30 minutes) at room temperature. Mononuclear cells were aspirated and the wall of the tubes was wiped to further remove adherent mononuclear cells. Neutrophils were then isolated from red blood cells by a step of sedimentation with 1% Dextran (GE Healthcare Life Sciences, Pittsburgh, PA) in 0.9% NaCl. Contaminating red blood cells were lysed under hypotonic conditions for 30 seconds. Purified neutrophils (95-98%) were washed twice with PBS and suspended in Hank’s balanced salt solution (HBSS) with Ca^2+^ and Mg^2+^ at pH 7.4 for microarray and respiratory burst analyses, or in RPMI 1640 medium containing 10% fetal bovine serum (FBS) for the cell adhesion assay.

**Neutrophil gene expression profiling using microarray.** After isolation, neutrophils were lysed using TRIzol reagent (Thermo Fisher Scientific) and frozen at -80°C. RNA was quantified using a ND-1000 NanoDrop spectrophotometer (NanoDrop Technologies, Wilmington, DE) and purity/integrity was assessed using disposable RNA chips (Agilent RNA 6000 Nano LabChip kit) in an Agilent 2100 Bioanalyzer (Agilent Technologies, Santa Clara, CA). Only RNA samples with RNA integrity numbers >8.9 were further processed for analysis. Clariom S Array, Human (Affymetrix) were hybridized at the genomic platform of the Curie Institute (Paris, France) according to manufacturer’s labelling and hybridization recommendations. This array was designed to measure the RNA expression of ~20,800 genes.

Analysis was performed as mentioned earlier for the whole-blood microarray data. Clariom S array dataset analysis and visualization were made using EASANA®, which is based on the GenoSplice’s FAST DB® annotations as described above for the whole-blood microarray data analysis.[4,5] Biological significance of changes across the 3 study groups (HS, AC and ACLF) was assessed using the Reactome database.

*Neutrophil adhesion assay*

Isolated neutrophils were suspended in RPMI 1640 medium containing 10% FBS and incubated for 30 min at 37°C. Neutrophils (1x10^6^/well) were then allowed to adhere for 30 minutes at 37°C to human umbilical vein endothelial cells (HUVECs, Huvec-c single donor ref C-12200) plated in 12-well plates precoated with gelatin and grown to confluence. After washing, cells (neutrophils and HUVECs) were harvested with trypsin-EDTA. CD66b^+^ (Beckman coulter ref PN-IM-0531U) polymorphonuclear neutrophils were counted using flow cytometry (LSR BD Biosciences) and expressed as a ratio of neutrophil per HUVEC.[10]

*Respiratory burst assays*

Respiratory burst was studied using the cytochrome c reduction assay , which is specific for superoxide anion.[11] Neutrophils were incubated at 37°C for 10 minutes in 1-ml plastic cuvette (1x10^6^ cell/ml HBSS). Cytochrome c (80 mM) was then added to the cell suspensions and the optical density (OD) at 550 nm was continuously monitored for 5 minutes (basal values) with a spectrophotometer (Perkin Elmer Lambda 40, Waltham, MA) equipped with a thermostatic chamber. Cells were then stimulated with N-Formyl-Met-Leu-Phe (fMLP, 1 µM) and the increase in cytochrome c reduction was continuously recorded. Total production of superoxide anion was calculated by subtracting basal values from those obtained at the plateau with fMLP. In some experiments, neutrophils were pre-treated with the TLR7/8 agonist CL097 (2 µg/ml) (Invivogen, San Diego, CA) for 10 min before stimulation with fMLP.

*Blood markers for inflammation, endothelial dysfunction and coagulation/platelet function*

Blood markers that were measured in the plasma included cytokines, chemokines and growth factors as well as endothelial and platelet activation factors. Measurements used multiplex immunoassays based on Luminex multi-analyte profiling technology (Human Cytokine/Chemokine Magnetic bead panel Premixed 38 Plex Kit and Human Sepsis Magnetic bead panel 1, Merk Millipore, Billerica, MA). Signals were read in a Luminex 100 Bioanalyzer (Luminex Corp., Austin, TX). A five-parameter logistic regression model was used to create standard curves and to calculate the concentration of each sample with the standard version of the Milliplex Analyst software (Merck Millipore). Of the 45 molecules that were assessed by the kits, 37 passed the filter of detection in 70% of samples. The plasma levels of two soluble markers of macrophage activation (soluble CD163 and soluble CD206) were determined by enzyme-linked immunosorbent assays (Antibodies-online, Aachen, Germany).

*Statistical analysis*

Quantitative variables were expressed as median (ranges) or mean (± standard deviation) where appropriate, and categorical variables as relative frequencies. Comparisons between groups of quantitative variables were performed using Mann-Whitney test. Comparisons between groups of qualitative variables were performed using chi-square test and the Fisher exact tests, as appropriate. All tests were two-sided and used a significance level of 0.05. Data handling and analysis were performed with SPSS 17.0 (SPSS Inc., Chicago, IL).

|  | | **Table S1. Study protocols according to the characteristics and origin of the enrolled participants.** | | | | | | | | | |  |
| --- | --- | --- | --- | --- | --- | --- | --- | --- | --- | --- | --- | --- |
| Study protocols | | | Characteristics of patients | | | | | | | | | |
|  | | | Patients with ACLF | | | Patients with acute decompensation | | | Patients with advanced cirrhosis | | Healthy subjects | |
|  |  |  | Number of patients | Patients with sepsis (%) | Median ACLF grade  (IQR) | Number of patients | Patients with sepsis (%) | Median CLIF-C AD score  (IQR) | Number of patients | Median MELD score  (IQR) | Number of participants | |
|  | | | **France** | | | | | | | | | |
| **First cohort** | | |  |  |  |  |  |  |  |  |  | |
| - Blood white-cell differential count analysis and whole-blood gene expression profiling using microarray | | | 17 | 47 | 3 (3-3) | 7 | 29 | 55 (52-59) | 7 | 10 (8-18) | 7 | |
| - Validation of microarray results using RT-qPCR | | | 11 | 64 | 3 (3-3) | − | − | − | 7 | 10 (8-18) | − | |
| - Measurement of 45 plasma inflammatory molecules | | | 16 | 44 | 3 (2.25-3) | - | - | - | − | − | 6 | |
| **Additional cohorts** | | |  |  |  |  |  |  |  |  |  | |
| - Analysis of blood immune cell subsets using flow cytometry | | | 7 | 57 | 3 (2.75-3) | − | − | − | 5 | 12 (11-18) | 7 | |
| - Neutrophil gene expression profiling using microarray | | | 5 | 60 | 2 (1.5-2.5) | − | − | − | 5 | 13 (8-21) | 5 | |
| - Assessment of CD177 expression at neutrophil surface using flow cytometry | | | 7 | 50 | 3 (2.75-3) | − | − | − | 5 | 12 (11-17) | 7 | |
| - Adhesion to HUVEC of neutrophil from patients with ACLF | | | 7 | 43 | 3 (2-3) | − | − | − | − | − | 7 | |
| - Measurement of superoxide anion production by neutrophils | | | 13 | 39 | 2 (1-2) | − | − | − | 13 | 23 (15-25) | − | |
| - Effect of CL097*^a^* on superoxide anion production by neutrophils | | | 8 | 50 | 2 (1-2.75) | − | − | − | − | − | − | |
|  | | | **UK** | | | | | | | | | |
| - Analysis of complete clinical blood counts | | | 203 | 30 | 2 (2-3) | 91 | 21 | 49 (43-54) | − | − | − | |
|  | | | **India** | | | | | | | | | |
| - Assessment of CD177 expression at neutrophil surface using flow cytometry | | | 12 | 100 | 2.5 (2-3) |  |  |  | 8 | 21 (18-23) | - | |
|  | NOTE: ACLF denotes acute on chronic liver failure, CLIF-C Chronic Liver Failure Consortium, AD acute decompensation score, IQR Interquartile range, MELD Model for End Stage Liver Disease, and RT-qPCR real time-quantitative polymerase chain reaction. | | | | | | | | | | | |
|  | *^a^* CL097 is an agonist of Toll-like receptor 7/8. | | | | | | | | | | | |

**Table S2. Definitions of the CLIF Consortium for organ system failures, acutely decompensated cirrhosis, and acute-on-chronic liver failure (ACLF) used in the present study.**

**Definitions of organ system failures***^a^*

Kidney: Creatinine ≥2 mg/dl or use of renal-replacement therapy

Lung: PaO_2_/FiO_2_ ≤200 or SpO_2_/FiO_2_ ≤214, or use of mechanical ventilation for other reason than hepatic encephalopathy (HE)

Liver: Total bilirubin ≥12 mg/dl

Coagulation: International Normalized ratio ≥2.5

Brain: HE Grade 3-4 in the West Haven classification or use of mechanical ventilation because of HE

Circulation: Use of vasopressors including terlipressin

**Definition of acutely decompensated cirrhosis***^b^*

Acute development of ascites, hepatic encephalopathy, gastrointestinal hemorrhage, or bacterial infection (in patients with prior AD), or any combination of these

**Definitions allowing patients stratification according to severity***^b^*

**Acute decompensation of cirrhosis (AD): Patients with acutely decompensated cirrhosis included in one of the following subgroups:**

(1) Patients with no organ failure

(2) Patients with single failure (affecting any of the following: liver, coagulation, circulation, respiration) and serum creatinine levels < 1.5 mg/dl without HE

(3) Patients with single cerebral failure and creatinine levels < 1.5 mg/dl

**ACLF: Patients with acutely decompensated cirrhosis included in one of the following 3 subgroups:**

ACLF grade 1 comprises:

(1) Patients with single kidney failure

(2) Patients with single liver, coagulation, circulatory or lung failure associated with creatinine levels ranging from 1.5 to 1.9 mg/dl or HE grade 1 or 2, or both

3) Patients with single brain failure with creatinine levels ranging from 1.5 to 1.9 mg/dl

ACLF grade 2 include patients with 2 organ failures

ACLF grade 3 include patients with 3 organ failures or more (maximum of 6).

NOTE: CLIF denotes Chronic Liver Failure, PaO_2_ partial pressure of arterial oxygen, FiO_2_ fraction of inspired oxygen, and SpO_2_ Pulse oximetric saturation.

*^a^* Definitions according to the CLIF-Consortium organ failure scoring system developed by Jalan, Saliba, Pavesi et al. [12]

*^b^* According to Moreau et al. [13]

| **Table S4. Characteristics of the English patients with acutely decompensated cirrhosis with and without ACLF, Related to Fig. S1.** | | | |
| --- | --- | --- | --- |
| Characteristic | Acute decompensation of cirrhosis (n=91) | ACLF (n=203) | *p* value |
| Median age (IQR) − yr | 52 (44-59) | 51 (41-60) | .76 |
| Male sex − n (%) | 61 (67) | 136 (67) | 1 |
| Etiology or cirrhosis |  |  | 0.17 |
| Alcoholic liver disease | 49 (53.8) | 121 (59.6) |  |
| Nonalcoholic steatohepatitis | 6 (6.6) | 2 (1) |  |
| Chronic hepatitis C | 8 (8.8) | 10 (4.9) |  |
| Chronic hepatitis B | 3 (3.3) | 12 (5.9) |  |
| Others | 25 (27.5) | 58 (28.6) |  |
| Precipitating events − n (%) |  |  | .08 |
| Sepsis | 19 (20.9) | 62 (30) |  |
| Excessive alcohol consumption | 9 (9.9) | 23 (11.3) |  |
| Hemorrhage | 45 (49.5) | 69 (34) |  |
| Other | 9 (9.9) | 2 (12.9) |  |
| None | 9 (9.9) | 33 (16.3) |  |
| Severity scores |  |  |  |
| Median CLIF-C AD score*^a^* (IQR) | 49 (43-54) | - | - |
| Median MELD score (IQR) | 15 (12-17) | 23 (18-29) | <.001 |
| Median CLIF-C organ failure score*^b^* (IQR) | 8 (8-9) | 12 (11-13) | <.001 |
| ACLF Grade*^c^* − n (%) |  |  |  |
| Grade 1 | 0 (0) | 25 (12.3) |  |
| Grade 2 | 0 (0) | 107 (52.7) |  |
| Grade 3 | 0 (0) | 71 (35.0) |  |
| Median ACLF Grade (IQR) | 0 | 2 (2-3) | <.001 |
| Median CLIF-C ACLF score*^d^* (IQR) | - | 54 (49-61) | <.001 |
| Median white-cell count*^e^* (IQR) - per mm^3^ | 8,110 (5,560-10,590) | 9,910 (6,310-15,855) | .001 |
| Median differential count*^f^* (IQR) − % |  |  |  |
| Neutrophils | 78 (70-83) | 82 (75-88) | .001 |
| Lymphocytes | 11 (8-15) | 8 (5-13) | <.001 |
| Monocytes | 8 (5-12) | 7 (5-10) | .29 |
| Median hemoglobin (IQR) − g/dL | 9.4 (8.0-10.2) | 9.1 (7.7-10.2) | .55 |
| Median platelet count (IQR) − per mm^3^ | 72,000 (47,000-105,000) | 77,000 (56,000-113,000) | .01 |
| Median International Normalized Ratio (IQR) | 1.6 (1.4-1.8) | 2.1 (1.7-2.7) | <.001 |
| Median serum sodium (IQR) − mmol/liter | 143 (138-146) | 138 (132-142) | <.001 |
| Median serum creatinine (IQR) − mg/dl | 0.7 (0.58-0.83) | 1.34 (0.84-2.06) | <.001 |
| Median total bilirubin (IQR) − mg/dl | 2.1 (1.3-4.0) | 6.0 (2.7-11.6) | <.001 |
| NOTE: *p* values were obtained using Mann–Whitney test and the chi-square test or Fisher’s exact test, as appropriate. ACLF denotes acute-on-chronic liver failure, IQR interquartile range, CLIF-C chronic liver failure-consortium, and MELD Model for End Stage Liver Disease. | | | |
| *^a^* The CLIF-C AD score, which takes into account age, serum creatinine, INR, white-cell count, and serum sodium, has been developed to predict death in patients with AD.[15] This score is only applicable to patients with AD. | | | |
| *^b^* The CLIF-C organ failure score includes subscores ranging from 1 to 3 for each of the six major organ systems (kidneys, lungs, liver, coagulation, brain, circulation). Organ system failures are defined in Table S1. Aggregated scores range from 6 to 18, with higher scores indicating more severe organ failure.[12] | | | |
| *^c^* ACLF grades are defined in Table S1. | | | |
| *^d^* The CLIF-C ACLF score, which takes into account the age, CLIF-C organ failure score, and blood white-cell count, has been developed to predict death in patients with ACLF. Scores of 70 or more indicate a very high probability of short-term death.[12] This score is only applicable to patients with ACLF. | | | |
| *^e^* Normal range for blood white-cell count is 4,500-11,000 per mm^3.^ | | | |
| *^f^* Normal ranges for differential counts are 40% to 70% for neutrophils, 4% to 11% for monocytes, and 22% to 44% for lymphocytes. | | | |

*^d^* Normal range for blood white-cell count is 4,500-11,000 per mm^3.^

*^e^* Normal ranges for differential counts are 40% to 70% for neutrophils, 4% to 11% for monocytes, and 22% to 44% for lymphocytes.

| **Table S9.** **Characteristics of the French patients with advanced cirrhosis (AC) and with ACLF in whom neutrophil transcriptome was investigated.** | | |
| --- | --- | --- |
| Characteristic | Advanced cirrhosis (AC, n=5) | ACLF (n=5) |
| Median age (IQR) − yr | 60 (49.5-68.5) | 54 (46-65) |
| Male sex – n (%) | 2 (40) | 3 (60) |
| Etiology or cirrhosis |  |  |
| Alcohol consumption | 5 (100) | 4 (80) |
| NASH | 0 (0) | 1 (20) |
| Sepsis | 0 | 3 (60) |
| Excessive alcohol consumption | 0 | 0 |
| Hemorrhage | 0 | 0 |
| Other | 0 | 2 (40) |
| None | 5 (100) | 0 |
| Severity scores |  |  |
| Median MELD score (IQR) | 13 (8-21) | 34 (27.5-37.5) |
| Median CLIF-C OF score*^b^* (IQR) | - | 12 (10-13.5) |
| Median CLIF-C Organ Failure subscores (IQR) |  |  |
| Kidney | 1 (1-1) | 1 (1-2) |
| Lung | 1 (1-1) | 1 (1-2) |
| Liver | 1 (1-1) | 3 (2-3) |
| Coagulation | 1 (1-2) | 2 (1-3) |
| Brain | 1 (1-2) | 2 (2-3) |
| Circulation | 1 (1-1) | 2 (1-3) |
| ACLF Grade*^c^* – n (%) |  |  |
| Grade 1 | 0 (0) | 1 (20) |
| Grade 2 | 0 (0) | 3 (60) |
| Grade 3 | 0 (0) | 1 (20) |
| Median ACLF Grade (IQR) | 0 (0-0) | 2.0 (1.5-2.5) |
| Median CLIF-C ACLF score*^d^* (IQR) | - | 67 (59-69.5) |
| Median white-cell count*^e^* (IQR) - per mm^3^ | 7,200 (4,900-7,850) | 13,300 (9,400-14,800) |
| Median differential count*^f^* (IQR) − % |  |  |
| Neutrophils | 66 (49-82) | 77 (70-81) |
| Lymphocytes | 9 (7-33) | 11 (4-16) |
| Monocytes | 14 (8-18) | 9 (5-12) |
| Median hemoglobin (IQR) − g/dl | 10.8 (9.9-17.8) | 8.6 (6.7-10.3) |
| Median platelet count (IQR) − per mm^3^ | 152 (87-187) | 56 (44.5-73.5) |
| Median International Normalized Ratio (IQR) | 1.18 (1.06-1.38) | 2.50 (1.9-3.7) |
| Median serum sodium (IQR) − mmol/liter | 133 (129-138) | 134 (127-141) |
| Median serum creatinine (IQR) − mg/dl | 0.96 (0.72-1.21) | 1.80 (1.50-3.40) |
| Median aspartate aminotransferase (IQR) − U/liter | 35 (23-56) | 158 (66-296) |
| Median alanine aminotransferase (IQR) − U/liter | 17 (13-26) | 55 (34-275) |
| Median total bilirubin (IQR) − mg/dl | 1.6 (0.8-2.8) | 26.6 (11.6-43.5) |
| NOTE: Neutrophil transcriptome was also assessed in 5 healthy subjects (2 males) with a median age of 57 years (Interquartile range [IQR], 23 to 65). *p* values were obtained using Mann–Whitney test and the chi-square test or Fisher’s exact test, as appropriate. ACLF denotes acute-on-chronic liver failure, IQR, interquartile range, CLIF-C, chronic liver failure-consortium, and MELD, Model for End Stage Liver Disease. | | |
| *^a^* The CLIF-C AD score, which takes into account age, serum creatinine, INR, white-cell count, and serum sodium, has been developed to predict death in patients with AD.[15] | | |
| *^b^* The CLIF-C organ failure score includes subscores ranging from 1 to 3 for each of the six major organ systems (kidneys, lungs, liver, coagulation, brain, circulation). Organ system failures are defined in Table S1. Aggregated scores range from 6 to 18, with higher scores indicating more severe organ failure.[12] | | |
| *^C^* ACLF grades are defined in Table S2. | | |
| *^d^* The CLIF-C ACLF score, which takes into account the age, CLIF-C organ failure score, and blood white-cell count, has been developed to predict death in patients with ACLF. Scores of 70 or more indicate a very high probability of short-term death.[12] | | |
| *^e^* Normal range for blood white-cell count is 4,500-11,000 per mm^3.^ | | |
| *^f^* Normal ranges for differential counts are 40% to 70% for neutrophils, 4% to 11% for monocytes, and 22% to 44% for lymphocytes. | | |

| **Table S11.** **Characteristics of Indian patients with advanced cirrhosis (AC) and with ACLF.** | | |
| --- | --- | --- |
| Characteristic | Advanced cirrhosis (AC, n=8) | ACLF (n=12) |
| Median age (IQR) − yr | 52 (46-57) | 43 (36-56) |
| Male sex – n (%) | 6 (75) | 5 (83) |
| Etiology of cirrhosis |  |  |
| Alcoholic liver disease | 5 | 7 |
| Nonalcoholic steatohepatitis | 3 | 2 |
| Chronic hepatitis B |  | 3 |
| Precipitating events – n (%) |  |  |
| Sepsis | 0 (0) | 12 (100) |
| Excessive alcohol consumption | 0 (0) | 0 (0) |
| Hemorrhage − % | 0 (0) | 0 (0) |
| Other − % | 0 (0) | 0 (0) |
| None − % | 8 (100) | 0 (0) |
| Severity scores |  |  |
| Median MELD score (IQR) | 21 (18-23) | 32 (29-37) |
| ACLF Grade*^a^* – n (%) |  |  |
| Grade 1 | 0 (0) | 0 (0) |
| Grade 2 | 0 (0) | 6 (50) |
| Grade 3 | 0 (0) | 6 (50) |
| Median ACLF Grade (IQR) | 0 | 2.5 (2-3) |
| Median white-cell count*^b^* (IQR) - per mm3 | 5,100 (2,500-10,875) | 10,250 (5700-21,200) |
| Median differential count*^c^* (IQR) − % |  |  |
| Neutrophils | 64 (59-74) | 75 (65-86) |
| Lymphocytes | 18 (11-21) | 10 (5-15) |
| Monocytes | 14 (11-18) | 10 (8-16) |
| Median hemoglobin (IQR) − g/dL | 9.1 (7.1-10.8) | 8.5 (7.4-10.3) |
| Median platelet count (IQR) − per mm3 | 73 (34-136) | 81 (32-109) |
| Median International Normalized Ratio | 1.6 (1.4-2.3) | 3.0 (2.5-3.5) |
| Median serum sodium (IQR) − mmol/liter | 136 (130-139) | 136 (130-139) |
| Median serum creatinine (IQR) − mg/dl | 2.3 (2.0-3.7) | 2.3 (2.0-3.7) |
| Median total bilirubin (IQR) − mg/dl | 0.8 (0.4-1.9) | 9 (5-15) |
| NOTE: ACLF denotes acute-on-chronic liver failure, IQR interquartile range, and MELD, Model for End Stage Liver Disease. | | |
| *^a^* ACLF grades are defined in Table S2. | | |
| *^b^* Normal range for blood white-cell count is 4,500-11,000 per mm^3^. | | |
| *^c^* Normal ranges for differential counts are 40% to 70% for neutrophils, 4% to 11% for monocytes, and 22% to 44% for lymphocytes. | | |

| **Table S12. Plasma levels of cytokines, chemokines, growth factors, markers of macrophage activation, coagulation/platelet function and endothelial dysfunction in healthy subjects, and patients with ACLF, all enrolled in France.** | | | |
| --- | --- | --- | --- |
| Variable | Healthy subjects  (n=6) | Patients with ACLF  (n=16) | *P* value |
| **Median values for cytokines (IQR) – pg/ml** |  |  |  |
| IL-2 | 0.5 (0.2-3.5) | 1.9 (1.0-3.9) | − |
| IL-4 | 0.13 (0.11-0.22) | 16.9 (10.6-26) | 0.001 |
| IL-7 | 0.6 (0.3-1.3) | 15.5 (9.0-31.6) | <0.001 |
| IL-15 | 2.0 (1.2-2.9) | 7.6 (5.6-23.8) | <0.001 |
| G-CSF | 4.9 (1.6-10.1) | 81.9 (27.9-292.3) | <0.001 |
| GM-CSF | 4.0 (0.7-7.8) | 14.4 (7.0-34.0) | 0.01 |
| IL-6 | 0.7 (0.3-1.3) | 182.6 (47.8-612.6) | <0.001 |
| IL-10 | 0.4 (0.2-1.0) | 43.6 (17.6-329.3) | 0.003 |
| IFN-α2 | 36.7 (13.5-50.4) | 39.5 (27.4-65.7) | − |
| IFN-γ | 18.5 (7.3-26.5) | 11.3 (4.5-25.7) | − |
| IL-1RA | 3.1 (2.3-5.6) | 80.0 (36.0-370.3) | <0.001 |
| IL-1α | 4.3 (0.7-5.9) | 79.0 (35.2-149.7) | <0.001 |
| TNF-α | 15.7 (5.6-21.1) | 29.4 (23.6-57) | 0.006 |
| Soluble FasL | 61.1 (36.8-75.8) | 28.4 (13.2-44.6) | 0.005 |
| TNFRSF6, also known as Fas | TLBD | 1,843 (1,642-2,623) | − |
| IL-17A | 4.7 (0.6-5.4) | 10.6 (4.7-32.3) | <0.05 |
| MIF | TLBD | 503.7 (152.3-1415.9) | − |
| **Median values for chemokines (IQR) – pg/ml** |  |  |  |
| MCP-1/CCL2 | 97.6 (76.0-115.2) | 899.1 (736.1-1215.0) | <0.001 |
| MIP-1α/CCL3 | 97.6 (76.0-115.3) | 22.8 (12.4-29.4) | 0.12 |
| MIP-1β/CCL4 | 9.7 (6.3-12.6) | 52.7 (34.4-59.5) | <0.001 |
| MCP-3/CCL7 | TLBD | 11.6 (8.8-24.7) | − |
| MDC/CCL22 | TLBD | 470.7 (299.9-718.3) | − |
| GROα/CXCL1 | TLBD | 1,269 (624-1,989) | − |
| IL-8/CXCL8 | 0.2 (0.5-0.7) | 228.4 (132.6-451.6) | <0.001 |
| IP10/CXCL10 | 111.1 (77.1-240.2) | 657.1 (360.7-1752.2) | 0.001 |
| Eotaxin | 46.7 (26.7-55.3) | 161.6 (115.1-217.9) | <0.001 |
| Fractalkine/CX3CL1 | TLBD | 73.8 (40.3-235.8) | − |
| **Median values for growth factors (IQR) – pg/ml** |  |  |  |
| EGF | 11.2 (3.2-29.1) | 43.6 (15.1-96.9) | 0.03 |
| FGF2 | TLBD | 88.3 (59.7-99.4) | − |
| VEGF | 22.9 (9.7-35.8) | 40.6 (19.8-109.6) | .1 |
| TGF-α | TLBD | 5.8 (3.6-11.5) | − |
| **Median values for markers of macrophage activation (IQR) – pg/ml** |  |  |  |
| Soluble CD163 | TLBD | 93.6 (66.7-9) | − |
| Soluble CD206 | TLBD | 17.1 (13.1-24.2) | − |
| **Median values for markers of coagulation/platelet function (IQR) – pg/ml** |  |  |  |
| tPAI-1 | TLBD | 31,238 (19,690-42,558) | − |
| sCD40L/TNFSF1 | TLBD | 1,011 (448-4,586) | − |
| **Median values for markers of endothelial dysfunction (IQR) – pg/ml** |  |  |  |
| Soluble ICAM-1 (pg/ml) | TLBD | 95,112 (67,210-149,287) | − |
|  |  |  |  |
| Soluble VCAM-1 (pg/ml) | TLBD | 293,318 (262,603-390,658) | − |
| TLBD denotes too low to be detected. |  |  |  |

**Table S13. Bone fide genes related to memory and exhausted CD8 T cell states, which are present or not among in the significantly differentially expressed genes between ACLF and HS, using whole-blood transcriptomic data (Table S4).**

| **Gene Symbol** | **Gene name** | **Regulation ACLF/HS (fold-change)** |
| --- | --- | --- |
| **Stem cell memory, central memory CD8 T cells** |  |  |
| *TCF7* | Transcription factor 7 (T-cell specific, HMG-box) | Down (2.70) |
| *LEF1* | Lymphoid enhancer binding factor 1 | Down (4.36) |
| *BCL2* | BCL2, apoptosis regulator | Down (3.16) |
| *IL7R* | Interleukin 7 receptor | Down (3.95) |
| *MYC* | V-myc avian myelocytomatosis viral oncogene homolog | Down (1.53) |
| *BACH2* | BTB domain and CNC homolog 2 | Down (2.09) |
| **Exhausted state** |  |  |
| *CTLA4* (also known as *CD152*) | Cytotoxic T-lymphocyte associated protein 4 | Not differentially expressed |
| *CD274* (also known as *PDL1*) | CD274 molecule | Not differentially expressed |
| *PDCD1* (also known as *CD279*, *PD1*) | Programmed cell death 1 | Not differentially expressed |
| *PDCD1LG2* (also known as *CD273*) | Programmed cell death 1 ligand 2 | Not differentially expressed |
| *VTCN1* (also known as *B7H4*) | V-set domain containing T cell activation inhibitor 1 | Not differentially expressed |
| *BTNL2* (also known as *BTL-II*) | Butyrophilin like 2 | Not differentially expressed |
| *TNFSF14* (also known as *HVEML*, *LIGHT*) | TNF superfamily member 14 | Not differentially expressed |
| *LAG3* | Lymphocyte activating 3 | Not differentially expressed |
| *TIGIT* | T cell immunoreceptor with Ig and ITIM domains | Down (1.97) |
| *HAVCR2* (also known as *TIM3*) | Hepatitis A virus cellular receptor 2 | Not differentially expressed |
| CD38 | CD38 molecule | Not differentially expressed |
| *ENTPD1* (also known as *CD39*) | Ectonucleoside triphosphate diphosphohydrolase 1 | Not differentially expressed |
| *BATF* | Basic leucine zipper ATF-like transcription factor | Not differentially expressed |
| *BTLA* (also known as *CD272*) | B and T lymphocyte associated | Down (2.29) |

**Fig S1.** Absolute counts of neutrophils, monocytes, and lymphocytes in AC, AD, and ACLF in the French cohort.

Kruskal–Wallis test was first used to compare the three groups. Mann–Whitney test was then used for pairwise comparisons. *: P <0.05, **: P <0.01, ***: P <0.001.

**Fig. S2.** Complete clinical blood count measurements in the English cohort of patients with acutely decompensated cirrhosis showed that ACLF associated with leukocytosis, neutrophilia, and lymphopenia.

Blood counts were measured in 91 patients with AD and 203 with ACLF. (A) Blood white-cell count. (B) Differential neutrophil, monocyte, and lymphocyte counts. (C) Correlation between differential neutrophil and differential lymphocyte count. P values were obtained using the Student’s *t-*test for unpaired data. **P <0.01.

**Fig. S3.** Unsupervised hierarchical clustering of whole-transcriptome measured in the 4 study groups including patients with advanced cirrhosis (AC), patients with acute decompensation of cirrhosis (AD), patients with ACLF, and healthy subjects (HS).

**Fig. S4.** Results of RT-qPCR measurements in a random set of genes using RNA extracted for whole-blood transcriptomics in French patients with advanced cirrhosis (AC) and those with ACLF.

(A) Relative expression of each gene by RT-qPCR in the comparison of ACLF versus AC (reference). (B) Results for the expression of corresponding genes in microarrays for the comparison of ACLF versus AC. The yellow color indicates the similarity of regulation of gene expression between the two techniques. (C) Primers used in this study. P values were obtained using Mann–Whitney test. ** P <0.01, * P <0.05.

B

| **Gene symbol** | **Expression levels** | | **Regulation (ACLF vs. AC)** |
| --- | --- | --- | --- |
|  | **AC** | **ACLF** |  |
| *IL6* | 4.71 ± 0.16 | 4.97 ± 0.14 | Not differentially expressed |
| *CXCL8* (also known as *IL8*) | 6.71 ± 0.51 | 6.89 ± 0.94 | Not differentially expressed |
| *IL10* | 4.64 ± 0.33 | 5.40 ± 0.53 | Not differentially expressed |
| *IL1B* | 8.55 ± 0.57 | 8.62 ± 0.90 | Not differentially expressed |
| *TNF* | 7.38 ± 0.25 | 7.11 ± 0.25 | down |
| *CXCL1* | 6.60 ± 0.36 | 6.50 ± 0.42 | Not differentially expressed |
| *CXCL2* | 5.21 ± 0.39 | 5.27 ± 0.20 | Not differentially expressed |
| *CXCL3* | 5.36 ± 0.14 | 5.62 ± 0.20 | Not differentially expressed |
| *HLA-DMA* | 8.57 ± 0.48 | 7.20 ± 0.71 | down |
| *HLA-DPB1* | 10.63 ± 0.47 | 9.04 ± 1.05 | down |
| *HLA-DRB5* | 10.26 ± 1.0 | 9.06 ±1.06 | down |
| *CIITA* | 8.34 ± 0.25 | 7.24 ± 0.55 | down |
| *CD74* | 11.22 ± 0.36 | 9.99 ± 0.81 | down |
| *CD4* | 9.65 ± 0.42 | 7.92 ± 0.96 | down |
| *CD9* | 7.35 ± 0.73 | 7.81 ± 0.64 | Not differentially expressed |
| *IRAK3* (also known as *IRAKM*) | 9.22 ± 0.60 | 10.88 ± 0.47 | up |
| NOTE: Plus–minus values are means ±SD. | | | |

**C**

| ***Genes (Aliases)*** | **Forward Primer** | **Reverse Primer** |
| --- | --- | --- |
| ***GAPDH*** | 5’ GCCCAATACGACCAAATCC 3' | 5’ AGCCACATCGCTCAGACAC 3' |
| ***IL6*** | 5’ CAATCTGGATTCAATGAGGAGAC 3' | 5’ CTCTGGCTTGTTCCTCACTACTC 3' |
| ***CXCL8 (IL8)*** | 5’ CACCGGAAGGAACCATCTCACTGT 3' | 5’ TCCTTGGCAAAACTGCACCTTCA 3' |
| ***IL10*** | 5’ GGCGCTGTCATCGATTTCTTC 3' | 5’ AGATGCCTTTCTCTTGGAGCTTATT 3' |
| ***IL1B*** | 5’ GCAAAAAAGCTTGGTGATGTCTG 3' | 5’ AAGGACATGGAGAACACCACTTGT 3' |
| ***TNF*** | 5’ GCCCAGGCAGTCAGATCATCTT 3' | 5’ CCTCAGCTTGAGGGTTTGCTACA 3' |
| ***CXCL1*** | 5’ TCGAAAAGATGCTGAACAGTGACA 3' | 5’ CTTCAGGAACAGCCACCAGTGA 3' |
| ***CXCL2*** | 5’ TCAAACCCAAGTTAGTTCAATCCTGA 3' | 5’ GCTGACATGTGATATGTCATCACGAA 3' |
| ***CXCL3*** | 5’ GGAGCACCAACTGACAGGAGAGAA 3' | 5’ ACCACCCTGCAGGAAGTGTCAA ' |
| ***HLADMA*** | 5’ TGTGGCAAGAAGGTATGGGT 3' | 5’ GTGTGCAGGAATGTGTGGTT 3' |
| ***HLADPB1*** | 5’ GCAGACACAACTACGAGCTG 3' | 5’ GTAGAAATCCGTCACGTGGC 3' |
| ***HLADRB5*** | 5’ CCTGGAGGTTCCTACATGGC 3' | 5’ AAGAAACGTGGTCGGGTGTC 3' |
| ***CIITA*** | 5’ GCTCTGGCAAATCTCTGAGG 3' | 5’ GGACGTCTTGTGCTCTGTCA 3' |
| ***CD74*** | 5’ TGACAGTCACCTCCCAGAAC 3' | 5’ TGCATCACATGGTCCTCTGT 3' |
| ***CD4*** | 5’ CTGAGTGACTCGGGACAGGT 3' | 5’ ATCTGAGACATCCGCTCTGC 3' |
| ***CD9*** | 5’ TTGGACTATGGCTCCGATTC 3' | 5’ CGGCTCCGATCAGAATATAGA 3' |
| ***IRAK3 (IRAKM)*** | 5' TGACAGATTGCAGTGTGTAGGTGA 3' | 5' GCA GGT AGT GAA TGG CTT TGG ATA 3' |

**Fig. S5.** Volcano plot of differential gene expression between patients with ACLF (n=17) relative to patients with advanced cirrhosis (AC, n=7).

Significance (−log_10_ P value) was plotted against the log_2_ fold-change (ACLF:AC ratio). Genes were considered as differentially expressed when P was <0.05 (or −log_10_ P >1.3; dashed horizontal line) and fold-change >1.5 (or log_2_ fold-change >0.58 for upregulation or log_2_ fold-change <-0.58 for downregulation [right and left dashed vertical lines, respectively]). Gray points indicate genes with no significant difference in expression, salmon indicates genes with significantly increased expression in patients and blue indicates genes with significantly decreased expression in patients. Representative differentially expressed genes are shown.

**Fig. S6.** Nine gene clusters capture the dynamics of gene co-expression across all study groups.

*(A)* Changes in the gene expression across study groups, for each gene of each of the 9 gene clusters we have computed. Each cluster was composed of at least 50 genes co-expressed across the patients’ groups relative to healthy subjects. For each module, the blue line indicates the expression of the eigengene that summarizes its behavior. (*B*) Histograms showing the values of each of the 9 eigengenes across the 4 study groups. Comparison between groups were performed using ANOVA. *(C)* Analysis of the enrichment of each gene cluster using the blood transcription modules (BTMs) developed by Li, Rouphael, Duraisingham, et al. [6] as gene sets. HS denotes healthy subjects, AC advanced cirrhosis, AD acute decompensation, and ACLF, acute-on-chronic liver failure.

**Fig. S7.** Analysis by flow cytometry of circulating monocyte subsets in healthy subjects (HS), patients with advanced cirrhosis (AC), and patients with ACLF.

Seven HS, 5 patients with AC, and 7 patients with ACLF admitted at Beaujon Hospital, Clichy, France, were investigated (Table S1). Monocytes were identified by forward and side scatter and combinations of CD14^+^ and CD16^+^. Kruskal–Wallis test was first used to compare the three groups. Mann–Whitney test was then used for pairwise comparisons. **: P <0.01

**Fig. S8.** Analysis by flow cytometry of circulating subsets of T cells, B cells and natural killer (NK) T cells in healthy subjects (HS), patients with advanced cirrhosis (AC), and patients with ACLF.

The same individuals as those studied for Fig. S5 were investigated in this analysis. Immune subsets were identified by forward and side scatter and combinations of the following markers: T cells (CD3^+^/CD4^+^ or CD3^+^/CD8^+^), B cells (CD19^+^), and NK T cells (CD3^+^/CD56^+^). P values were obtained using the Kruskal-Wallis test and the Mann-Whitney test. *: P <0.05, **: P <0.01, ***: P <0.001.

**Fig. S9**. CD177^+^ neutrophils in blood from Indian patients with advanced (AC) cirrhosis and patients with ACLF.

Eight patients with AC and 12 with ACLF, all admitted in the Department of Hepatology, Institute of Liver and Biliary Sciences (ILBS) New Delhi, India, were investigated. Flux cytometry analyses were performed in the Department of Research at ILBS. Neutrophils were identified by forward and side scatter and the following combination of markers: CD15^+^/CD16^+^/CD33^-^. P values were obtained using the Mann-Whitney test. **P: <0.01.

**Fig. S10.** Mean Florescence Intensity (MFI) of CD177 in blood neutrophils from French individuals including healthy subjects (HS; n=7), patients with advanced cirrhosis (AC; n=5), and patients with ACLF (n=5).

Investigations have been performed in the same individuals as for Fig. 4D. Kruskal–Wallis test was first used to compare the three groups. Mann–Whitney test was then used for pairwise comparisons. *: P <0.05.

**References**

[1]	Chiche A, Di-Cicco A, Sesma-Sanz L, Bresson L, de la Grange P, Glukhova MA, et al. p53 controls the plasticity of mammary luminal progenitor cells downstream of Met signaling. Breast Cancer Res 2019;21:13.

[2]	Chapat C, Chettab K, Simonet P, Wang P, De La Grange P, Le Romancer M, et al. Alternative splicing of CNOT7 diversifies CCR4-NOT functions. Nucleic Acids Res 2017;45:8508-8523.

[3]	de la Grange P, Gratadou L, Delord M, Dutertre M, Auboeuf D. Splicing factor and exon profiling across human tissues. Nucleic Acids Res 2010;38:2825-2838.

[4]	de la Grange P, Dutertre M, Martin N, Auboeuf D. FAST DB: a website resource for the study of the expression regulation of human gene products. Nucleic Acids Res 2005;33:4276-4284.

[5]	de la Grange P, Dutertre M, Correa M, Auboeuf D. A new advance in alternative splicing databases: from catalogue to detailed analysis of regulation of expression and function of human alternative splicing variants. BMC Bioinformatics 2007;8:180.

[6]	Li S, Rouphael N, Duraisingham S, et al. Molecular signatures of antibody responses derived from a systems biology study of five human vaccines. Nat Immunol 2014;15:195-204.

[7] Newman AM, Liu CL, Green MR, et al. Robust enumeration of cell subsets from tissue expression profiles. Nat Methods 2015;12:453-457.

[8]	Gandoura S, Weiss E, Rautou PE, Fasseu M, Gustot T, Lemoine F, et al. Gene- and exon-expression profiling reveals an extensive LPS-induced response in immune cells in patients with cirrhosis. J Hepatol 2013;58:936-948.

[9]	Rolas L, Makhezer N, Hadjoudj S, El-Benna J, Djerdjouri B, Elkrief L, et al. Inhibition of mammalian target of rapamycin aggravates the respiratory burst defect of neutrophils from decompensated patients with cirrhosis. Hepatology 2013;57:1163-1171.

[10]	Marino F, Schembri L, Rasini E, Pinoli M, Scanzano A, Luini A, et al. Characterization of human leukocyte-HUVEC adhesion: Effect of cell preparation methods. J Immunol Methods 2017;443:55-63.

[11]	Casini A, Ceni E, Salzano R, Biondi P, Parola M, Galli A, et al. Neutrophil-derived superoxide anion induces lipid peroxidation and stimulates collagen synthesis in human hepatic stellate cells: role of nitric oxide. Hepatology 1997;25:361-367.

[12]	Jalan R, Saliba F, Pavesi M, et al. Development and validation of a prognostic score to predict mortality in patients with acute-on-chronic liver failure. J Hepatol 2014;61:1038-1047.

[13]	Moreau R, Jalan R, Gines P, et al. Acute-on-chronic liver failure is a distinct syndrome that develops in patients with acute decompensation of cirrhosis. Gastroenterology 2013;144:1426-1437, 1437 e1421-1429.

[14] Trebicka J, Fernandez J, Papp M, et al. The PREDICT study uncovers three clinical courses of acutely decompensated cirrhosis that have distinct pathophysiology. J Hepatol 2020, in press.

[15]	Jalan R, Pavesi M, Saliba F, et al. The CLIF Consortium Acute Decompensation score (CLIF-C ADs) for prognosis of hospitalised cirrhotic patients without acute-on-chronic liver failure. J Hepatol 2014.
